# Quarterly Periscope of Practical Medicine; Being *the Spirit of the Medical Journals*, Foreign and Domestic

**Published:** 1826-10-01

**Authors:** 


### Quarterly Periscope OF PRACTICAL MEDICINE;

BEING

### The Spirit of the Medical Journals,

*Foreign and Domestic;*

WITH COMMENTARIES.

---

"Neglecta reducit, sparsa colligit, utilia selegit, necessaria ostendit,—sic utile."—BAGLIVI.

---

THE motto which we have taken for our Periscope, from the illustrious Baglivi, is sufficiently indicative of the principles which govern us in its construction. No source of useful information, foreign or domestic, shall be overlooked in our search after truth and facts, so as to comply with the mandate, "sparsa colligit." But to fulfil the difficult task of "utilia selegit," we must be allowed some discretionary power. Numerous are the articles which come abroad into the medical world, under imposing titles, and dressed in the specious garb of truth, which the eye of experience soon discovers to be spurious or counterfeit; and of which we could offer many curious but perhaps invidious specimens. We choose rather to pass them unnoticed, though at the risk of being thought negligent by some. The precept "necessaria ostendit," requires equal care and discrimination with that of "utilia selegit."

——————— Pauci dignoscere possunt
Vera bona.

In our endeavours to accomplish this important part of our duty, we aspire not to infallibility, and need the indulgence of the Public; for to give entire satisfaction to *all*, is impossible. Since our last publication, however, in which was announced the intention of the Medical and Physical Journal to commence a series of AUTHENTICATED HOSPITAL REPORTS, we have received several communications from town and country, respecting this subject; all concurring in the suggestion of dedicating a separate section in the Periscope to an Analytical *Review* of such well-attested records of hospital practice as appear in the periodicals of this and of other countries. We readily acquiesce in the utility of such a plan, and have, this quarter, put it in operation. In our next number we hope to make the arrangement still more complete, and to be able to incorporate with these Reports (in the shape of commentaries or otherwise) some important practical information derived from sources the most authentic. In the mean time, we reiterate our exhortation to the medical officers of hospitals, metropolitan and provincial, to follow the example of those distinguished individuals who have already commenced their clinical reports in our respected cotemporary, the MEDICAL and PHYSICAL JOURNAL. In reviewing these reports, and collocating them with similar documents from foreign hospitals, we hope to give encouragement as well as an extension to their circulation that may prove equally advantageous to the contributors and to the profession at large—and that, in so doing, we shall fulfil the last object contained in our motto—"sic utile."

The commercial difficulties of the times having borne with more than proportionate weight on the press, in this country, the publication of valuable medical works has been greatly crippled, and the periodical journals and transactions are now the principal channels through which medical men can come before the public, without considerable pecuniary loss. This circumstance will, in all probability, increase the afflux of important communications to those useful vehicles of information, and render them the depositaries of numerous facts and observations which, in more prosperous times, would be spread out into costly monographs. But, as a portion (and not a small portion) of the general embarrassment comes home to the door of almost every individual of the medical profession, it rarely happens, especially in the country, that any individual can afford to take in more than one, or at the most two, periodical journals. On this account, it will turn out to be the true interest of medical journalists to adopt, as far as possible, that division of labour which is found to succeed best in all other departments of art, science, or literature. If two or more periodical works run upon precisely the same plan, and contain nearly the same species of materials, it is evident that a principle of selection will be acted upon by the purchasers, and that consequently some, or indeed all must thereby suffer. This is more particularly the case with respect to REVIEWS. There is but a limited range of monographical publications in medicine, and if there were fifty medical reviews, they can *all* give but accounts of the *same* works. That Review then will be selected which is thought to give the best delineation of the works reviewed; for, in medical science, the plan adopted in general literature, of making the titles of a leash of books the peg on which to hang a critical dissertation, cannot, for obvious reasons, be adopted. If, therefore, one journal confines itself principally to original communications—another to hospital reports—a third to analyses of books, &c. there will be a fairer chance for all to succeed. For, if one individual cannot afford to take in more than one work, he will club with his neighbour or neighbours, and they will take in two or three, if the works are conducted upon different plans, and contain different kinds of information. The truth of these remarks must indeed have been forcing itself upon the observation of journalists for some time past; and we see it unequivocally operating upon their conduct already. Thus the Edinburgh Medical and Surgical Journal has long been conspicuous, and in the highest esteem, for the number and extent of its original communications; and our respected contemporary, the London Medical and Physical Journal, has now adopted a plan of publishing authenticated hospital reports, which has already increased, and will still farther increase its wide circulation and long-established celebrity. There is sufficient field for ingenuity and talent to strike out into yet other plans for exercising the intellect, augmenting the stock of knowledge, and diffusing information through the various ramifications of medical society. We wish them all success.

1. ON GASTRALGIA, MISTAKEN FOR GASTRITIS. BY DR. BARRAS.*

Medical men, of the new light, both on the Continent and in this country, commit daily blunders, and put their patients to great sufferings, by confounding gastralgia with gastritic affections—in other words, the neuroses with the phlogoses. We know this by numerous observations —and we know it to our cost. We have gained useful experience on this point, and we most seriously recommend a very careful perusal of the following memoir, not only to young practitioners, but to the whole host of writers on gastric, hepatic, and dyspeptic derangements. It may save many thousand leeches—many pounds of blue pill—and, what is of more consequence, many painful days and sleepless nights, to their patients!

Dr. Barras hesitated long before he brought himself to lay before the public the particulars of his own case—partly from shame for having been so much in error himself—but principally for fear of hurting the feelings of some eminent physicians, under whose advice he had acted. A sense of duty to the cause of truth and science has overcome these scruples, and it would be well for the *latter* in particular, if more disclosures of this kind were brought before the eye of the profession through the medium of the public press. The reader will please to excuse both the patient and ourselves, if we be somewhat more particular in the detail of history and symptoms, than may, at first sight, appear necessary.

*Case.* M. Barras is now in the 46th year of his age. When 24 years old, and then an intern student in the St. Louis Hospital, he became affected with a violent neuralgia of the right temple. This pain, which was unaccompanied with any febrile phenomenon, commenced at 10 o'clock every morning, and lasted about two hours. It was aggravated by antiphlogistic remedies, and suddenly gave way to a blister to the nucha. In the 29th year of his age, he began to experience a neuralgic affection of the spermatic cord, which harrassed him for the space of four years, and the history of which was published in the *Bibliothèque Medicale* for 1813. This complaint finally gave way to the repeated applications of moxa to the seat of the pain—that is, immediately below the inguinal ring. On this occasion our author had an opportunity first to remark a phenomenon which has often presented itself to him subsequently—namely that, in the neuralgiæ, the moxa, when applied to the *nerve actually affected*, does not increase the sufferings, but produces a peculiar sensation which gradually spreads to the various ramifications of the said nerve;—whereas, if applied to a part at some distance from the *actual* seat of the neuralgia, it greatly aggravates the pain ("il fait horriblement souffrir") and the patient experiences no benefit from the measure. In March 1815, then 36 years of age, and when exposed to some severe moral afflictions, M. Barras became affected

* Sur des Gastralgies Nerveuses Hypochondriaques, prises pour Gastro-enterites Chroniques. Par M. Barras, M. D. Revue Medicale, Nov. et Dec. 1825.

with a species of irregular intermittent, the accessions of which took place two or three times in the 24 hours, consisting of a severe pain over the right eye, accompanied by a spasmodic cough. In about a fortnight these symptoms were complicated with some acceleration of pulse and heat of skin, terminating in a copious perspiration. There were no rigors, and the appetite remained unaffected. The patient continued to attend to his affairs, and neglected his complaint till the end of April, when he consulted an eminent physician, who, after a patient examination, prescribed an emetic, and afterward the bark in substance, with light nourishing diet. The cinchona would not lie on the stomach, and the complaint continued without diminution. A blister to the nucha, however, removed the pain of the eye-brow, the other symptoms remaining in *statu quo*. The cough continued so obstinate, and was accompanied by so much emaciation, that apprehensions of phthisis were entertained. He was, therefore, sent into the country, and all medicine was discontinued, on the 12th July. In the course of eight days the febrile phenomena disappeared—he began to gain strength and flesh—and by the end of August he returned to Paris, with only a slight cough. For this he was recommended to live a good deal on grit-gruel with milk—a diet which, being long continued, he accuses of occasioning much subsequent suffering, from its debilitating effects on his stomach. His digestion now became gradually weak, imperfect, and painful, accompanied by a sense of weight at the epigastrium, pains in the pectoral muscles, variable appetite—eructations, flatulence, colic, constipation. These symptoms were all exasperated in damp and hot weather with southerly winds, and ameliorated by contrary states of the atmosphere—especially by an excursion of even a few days into the country. A complete gastric affection became now established by degrees—it might be called an *acute* supervening on a *chronic* gastralgia —"for the neuroses, like the phlegmasiæ, may be either chronic or acute"—an observation in which we entirely agree, though it is little attended to by physicians. An exasperation was caused early in 1823, by severe studies and anxiety of mind. To the symptoms enumerated above, were added a violent pain in the epigastrium, of a kind which requires particular notice. It always began about two or three hours after meals—at first by a sense of constriction in the stomach, then pain, and ultimately nausea, and an indescribable malaise. The digestion finished, these symptoms all disappeared, to be renewed each time that food was introduced into the stomach. Although M. Barras continued his professional avocations, he wasted in flesh, and as he now feared he had a chronic gastro-enteritis, he applied to the physician who first attended him for the intermittent. This gentleman affirmed that the complaint was not a gastro-enteritis, but he gave him to understand there was considerable danger attending it, which declaration occasioned the most poignant alarm in the patient's mind, and produced bad consequences in the end. For although the plan recommended by this physician (magnesia, the waters of Vichy—mild animal food, &c.) was attended with great amelioration of the symptoms, still the patient's mind was haunted with apprehensions for the future. Soon after returning to the capital a relapse took place. The sight of patients, especially those who had any gastric affection, induced the most lively agitation of mind. These circumstances, together with the intense heat of August and September, threw the patient as far back as ever. He now applied to another physician who, being inclined to Broussaisism, pronounced the disease at once to be a "*gastro-enterite*." It was in vain that M. Barras alluded to the whiteness of his tongue, the absence of fever, thirst, or tenderness on pressure of the epigastrium—the habitual constipation, &c. The disease was gastro-enteritis. The tongue *would* become red—fever *would* arise by and bye—diarrhœa *would* supersede constipation. The treatment is easily imagined. Leeches to the epigastrium—slops for food—and simple water for drink. Country air indeed was added, which was the wisest part of the advice. The remedies recommended aggravated every day the complaint. Even the country air this time failed in its usual beneficial effects, and when the gloomy month of November set in—then agitation, despondency, sleeplessness, and tædium vitæ reigned triumphant over the unhappy patient! The sensibility of the stomach now seemed to acquire a new degree of intensity. "D'organique elle devint animale—pour me servir du langage de Bichât." The stomach also became the seat of the most strange and anomalous sensations, sometimes of burning heat—sometimes of icy coldness—sometimes of formication, as if animals were creeping about in it. The pain a few hours after eating now became insufferable—in short, the stomach seemed now incapable of *bearing* the presence of any alimentary substance, without extreme pain, ending in nausea and the extrication of large quantities of gas, after which a respite from suffering ensued. Under these circumstances it was remarked by the patient, that liquids caused greater gastralgia than solids; and yet, notwithstanding all these indications, M. Barras persevered most resolutely in the watery regimen. But things at length arrived at such a crisis, that it was with the utmost difficulty he could resist the propensity to suicide. Mean-while some new phenomena were added to those already on record. M. Barras became extremely sensible to external cold—his feet were like lumps of ice—he felt strange sensations in different parts of the body—and the propensity to make water (which was as clear as from a fountain) was incessant. Palpitations of the heart were now distressing, as well as the most extraordinary pulsations in all tangible arteries. To these were at length added two or three daily febrile accessions, consisting merely of acceleration of pulse, heat of skin, and ultimate perspiration. This last train of phenomena completed our author's fears, or rather his hopes, of approaching death, since it had been predicted that *fever* would at last arise, when the disease had got to its height.*

* This is an admirable picture of the state to which a patient with *dyspepsia* may be reduced by the antiphlogistic regimen. The writer of this article was once nearly destroyed by following the advice of a modern

M. Barras' relations now in despair brought another physician, a pure Broussaian, who not only recognized *chronic*, but a severe attack of *acute* gastritis, which had supervened on the other! Nothing short of 40 leeches to the epigastrium could be of any use. M. Barras refused at first, but overcome by the entreaties of his family and his physician, he assented. This measure nearly completed the business. Rapid marasmus—vomitings—faintings—excessive flatulence—utter inability to bear any food—dreadful pain, &c. promised soon to put a period to the unhappy and misguided sufferer's life and ailments! We now hasten to a close. In this dreadful condition M. Fouquier was called in, and after an attentive examination of the history and symptoms, he pronounced the following sentence, in the truth of which we entirely agree. "You have no inflammation, said he—you never had any. The complaint is a gastralgia, an excess of sensibility in the nerves of the stomach, and nothing more."—"The treatment," M. Fouquier continued, "is very simple, and success is certain." These consolatory expressions alone had a considerable effect in mitigating the symptoms. The regimen is easily imagined. Light animal food instead of slops and gum-water:—Bourdeaux wine was substituted for the crystal spring—the cold bath was ordered, and a blister to the epigastrium. Still, it was some time before the stomach would bear proper aliment, after the long debilitation which an erroneous view of the case had produced in that organ.

It is a curious circumstance that, about this time, a severe domestic affliction (a fatal illness of M. Barras' daughter) contributed greatly to his own cure, by attracting all his anxiety to the dangerous state of his child, and withdrawing his reflexions from his own complaint.

The above case is exceedingly instructive, and it is one of very common occurrence in these days of hobby-horsical theories, when every pain in the stomach or imperfection in the process of digestion is attributed to chronic inflammation of the mucous membrane of the stomach or duodenum, and when leeches, blue pill, ipecacuan and sarsa, are the only remedies which can enter the minds of the digestive organists, while the wretched patient is put upon a slop and vegetable diet which aggravates the disease. The delusion is increased by patients evincing tenderness when pressed hard in the epigastric region. We can assert from numerous observations, and from personal suffering, that there is more tenderness in these states of neuralgic affection than in the opposite conditions of chronic inflammation. No one symptom, therefore, should be trusted to, but a careful examination made of the whole phenomena.

But it may be said that an insulated case is nothing to draw a conclusion from. This is true. And yet one well authenticated fact is often sufficient to upset a fine-drawn theory. But M. Barras does not

---

physician on this point. So far from there being any inflammatory diathesis in the above case, the whole phenomena depended on an opposite condition of the stomach—*atony*.—*Rev.*

rest his conclusions on isolated cases, or exceptions to general rules. He supports his subject by many facts. Three cases of hypochondriac gastralgia are recorded by M. Georget, (whose veracity is unimpeachable) where the antiphlogistic treatment produced corporeal extenuation and a kind of mental imbecility. Of two similar cases that came under M. Barras' observation, one terminated fatally—and this case he now publishes, to clear his conscience.

A young lady having, in the year 1793, experienced great moral affliction, fell into a state of melancholy, attended with pains in the epigastric region—aggravated after eating. During the many years which this malady continued, she consulted numerous physicians, but without any permanent cure. She had occasional and long intervals of comparative ease, but never a total cessation of the complaint. The least error in regimen or disagreeable moral emotion was sufficient to renew the disease at any time. In the year 1819, a combination of circumstances had caused aggravation of the complaint, and it was at this period, (the patient being in her 47th year) that M. Barras was consulted. She now complained of eructations, nausea, constipation. Immediately after eating, she felt as if there was a lump of lead in her stomach, which sensation went off, when the gastric digestion was finished. There was, in this case, no tenderness on pressure of the epigastrium. She was not emaciated nor reduced in muscular strength. She felt, however, an unaccountable apathy and nonchalance. Imbued at that period with the new physiological doctrine, and convinced that the disease was a chronic gastro-enteritis, M. Barras ordered, of course, the antiphlogistic treatment—but in a moderate degree, applying only 16 leeches to the epigastrium at a time.—But the malady made progress, in spite of the leeches, and so quickly too, that our author requested a consultation. The physician who was called in, participated in the erroneous opinion with M. Barras. They prescribed Seltzer water, which was followed by enormous tympanitis that resisted every remedy, and the poor lady died in dreadful torments. Examination of the body was not allowed; but M. Barras is now fully convinced that gastro-enteritis was not the disease which caused the death of the patient.

*Case.* Madame Capdeville, aged 56 years, had been subject to much mental sufferings, and constantly complained of pain in the region of the stomach. She was treated as for gastro-enteritis of a chronic kind; but died rather suddenly in the month of June, 1822. M. Barras was called to examine the body, and the physician who had the charge of the patient, pronounced, with all the confidence of a true disciple of Broussais, that they would find the "inevitable" cause of all diseases—inflammation, if not ulceration, of the mucous membrane of the stomach and bowels. Nevertheless, this membrane was found perfectly natural, both in the stomach and intestines, with the exception of a slight blush of redness on a small patch of the ileum. There was some serous effusion in the ventricles of the brain, and a slight enlargement of the heart.

The next case related was not quite so melancholy. The vigour of the patient's constitution happily triumphed over both the malady and the remedies.

*Case.* A man, upwards of 30 years of age, clerk in a Notary's office in Paris, was disposed to gastralgia, in consequence of sedentary habits and hard labour at the desk; the disorder having been greatly aggravated by chagrin at the death of his mother. The complaint commenced, as it usually does, with difficult digestion and pains in the epigastrium, exasperated after eating, and mitigated when the stomach was empty. A young physician, a disciple of Broussais, was consulted, and it is hardly necessary to say that he instantly detected a chronic "*gastro-enterite.*" After 120 leeches had been applied in different squadrons, and the usual antiphlogistic remedies had been employed, in conjunction with starvation, the malady, instead of giving way, had considerably increased. The pains in the stomach were much more severe, and greatly exasperated by the smallest quantity of ingesta, which also produced eructations and nausea. Constipation became obstinate—the abdomen was generally distended with gas—*urine pale, and passed in large quantities*—strength and flesh decreasing rapidly—palpitations and breathlessness on using any exertion. To these symptoms were added, morbid sensibility to cold—mental irritability—despondency—ennui, and disgust of life. The patient had been eleven months in this condition, when M. Barras was requested to see him, and found him as above described, the emaciation having arrived to an actual marasmus—and the tædium vitæ threatening suicide daily. On examination, he was convinced there was no organic disease of the stomach—no chronic inflammation of the intestinal tube—in short, that the disease was of the class neuroses. After encouraging the patient as much as possible by assurances of recovery, M. Barras discarded at once the warm baths, the debilitating slops, and the other parts of the antiphlogistic regimen—prescribing the reverse, but beginning cautiously, of course, and warning the patient that his stomach would find considerable difficulty at first, in overcoming animal food. The advice of M. Barras was but partially followed for a time, on account of the fears of the patient, and his ordinary medical attendant dreamt of nothing but *gastro-enterite.* Nevertheless a gradual improvement took place—the patient gathered courage—undertook a journey into the country—and returned to the capital in perfect health.

Such is the delusion of the Broussaian disciple, that he persists to this day in averring that it was his plan, so long previously pursued, which cured this patient of the "*gastro-enterite.*" Thus, if the patient dies, the advocate of Broussais exclaims that the disease was incurable.—If his constitution resists the malady and the remedies, then he swears he cured him—if he gets worse on slops, and recovers on solid food, the slops were the sole cause of the fortunate event!—The same thing is daily occurring in this country. Tenderness on pressure in the epigastrium is the signal for leeches, blue pill, and slops. The patient gets worse, and the remedies are increased. At length, tired out, the patient escapes into the country and recovers. The doctor takes all the credit of curing the patient, who only wanted a little country air to complete his convalescence! Aussi va le monde.

If you shew the disciple of Broussais or his prototypes here, the mucous membrane of a patient, who dies of this complaint, as white as a piece of boiled tripe, he begins to scrape it with his scalpel and at length discovers, or produces, some blush of redness in the part. His triumph is then complete. If he cannot do this, then the gastro-enterite has disappeared in death—a most convenient doctrine for the school of Broussais!

It is with the disciples of Broussais in France, and the digestive-organists in this country, as it was formerly with the disciples of the celebrated Corvisart. It is well known that when that eminent physician first published his work on diseases of the heart, half the students in France fancied themselves ill with organic affection of the central organ of the circulation. They felt their own pulses, and the mental emotion caused the heart to beat quick or even irregular. This was enough. All was lost. Bleeding and starvation increased the derangement of function in the circulating system, and as many of them died as could be killed by the force of imagination! So with the blind followers of Broussais, Abernethy, and others:—they expose their tongues to every looking-glass—and if there be any fur at the root, or redness at the tip—they have the "inevitable *gastrite ou duodenite chronique.*"—Leeches and slops render the stomach so weak and irritable that, when they attempt any solid food, they are greatly distressed—and thus they are confirmed in their first erroneous impressions.

*Case.* A butcher in Paris, whose father had died at the age of 42 years, of scirrhus of the pylorus, became persuaded that he had the same disease, whenever he felt any uneasiness in the stomach. In March, 1825, his imagination had so aggravated his feelings that the stomach was tormented with severe gastralgia, nausea, and even vomiting. M. Barras was consulted, and soon discovered that the disease was gastralgia, and not gastritis. By assuring the patient that there was nothing like cancer of the stomach in his case, he took courage, was put on a proper regimen, and soon got quite well.

About six years ago our author was consulted by a lady, of middle age, on account of a hypochondriacal gastralgia which had come on after some domestic affliction. Having gone for some time into the country, the lady consulted, in his absence, two celebrated physicians, who pronounced the disease "*une superbe gastro-enterite chronique,*" which would be cured in six weeks by relays of leeches to the epigastrium, gum water, milk diet, and the warm bath. The means were used, but the prediction was not verified. At the end of six weeks she was worse than at the beginning. The disease has continued, because the moral causes that first produced it are still in operation; but there is no reason to believe there is any organic, or even inflammatory affection of the stomach.

There is an American gentleman now in Paris, who came over to be cured of a gastralgic affection which he has laboured under for the space of 25 years. During the last 22 months the complaint has been attended with daily vomiting. He has been under more than a dozen of physicians, who have treated him, some for an organic disease, some for chronic gastritis, some for neuralgia of the stomach. None of them have succeeded;—and for this reason, that the patient himself is convinced the villous coat of the stomach is destroyed, and that the disease is incurable. This infatuation keeps up the complaint. He constantly uses the cold bath, and in that only finds relief. He lives almost entirely on sugar. Yet he preserves his strength and flesh, and his complexion is perfectly clear and healthy. "Is it possible that this case can be gastritis? No:—if it were, he would have been dead long ago." There is this essential difference between gastritic and gastralgic affections—that the *former* is soon (comparatively speaking) fatal; whereas the *latter* seldom destroys life unless very improperly treated.

GENERAL AND COMPARATIVE HISTORY.

I. The pain, in *chronic gastro-enteritis*, is generally obtuse, as is the case in all inflammations, whether acute or chronic, of the mucous membranes. This pain is often only felt on pressure—but whatever be its degree of intensity, it is never absent, from the beginning till the end of the complaint. Gastralgic or nervous pain, on the other hand, is frequently of extreme violence, and what is remarkable, it is often at these times relieved rather than increased by firm pressure. This pain, which occasionally radiates from the epigastrium towards the thoracic parietes, the back, and the shoulders, is of an intermittent character, sometimes entirely disappearing, to return again with more or less violence.

II. In *chronic gastritis*, the tongue, which is generally red on the sides and at the tip, is covered in the middle with a kind of dry mucous crust, resembling a false membrane—the breath is fetid—with a bitter taste in the mouth—there is thirst. In *gastralgia*, the tongue is white—saliva abundant—no thirst, but sometimes a repugnance even to liquids.

III. In *gastritis*, the appetite is always bad, and sometimes amounts to a universal disgust towards every kind of food. In *gastralgia*, the appetite is variable—null—slight—natural—often greater than in health.

IV. In *chronic gastritis*, the ingestion of a small quantity of food renews the patient's sufferings—excites a febrile movement in the system—and the digestion is always imperfect.—There is often rejection of the food by vomiting a little time after eating—or, if there be no vomiting, the patient is oppressed during the digestive process, with a sense of weight, distention, nausea, acid or acrid eructations, and irritation of the bowels or diarrhœa in the advanced stages. In some cases of *gastralgia*, the pain is relieved, at least for a time, by eating food in considerable quantity, and the digestion is complete, or even too quick. In the generality of cases, however, of gastralgia, the presence of food in the stomach renews the pain; but not till some time after eating; generally one, two, or even three hours; at which time the patient experiences weight and malaise at the epigastrium, as if there was a foreign body in the stomach. There are nausea, borborygmi, flatulent colic, eructations of air, but without fetor or causticity. Sometimes, indeed, they will taste the aliments that they have swallowed in the air which they eructate, but the digestion is completed, and diarrhœa is very rare. Constipation, indeed, is generally obstinate—and the urine, especially when the gastralgia is in a high degree, is usually pale, rendered frequently, and in small quantities at a time.

V. The chronic gastrites, however slow in their march, never fail in the end to exert a morbid influence on the process of nutrition, inducing hectic fever, characterized by hardness and frequency of the pulse, heat of skin, and evening exacerbation, loss of flesh and strength, sallowness of countenance, with a peculiar dark tinge—and lastly, death, after the resources of art have been exhausted in vain!

We see, on the other hand, people complain for ten, fifteen, twenty years—nay, the whole of their lives, of gastric pains, without fever, loss of flesh, or diminution of strength. Such cases, it is true, are comparatively rare; for, in general, a continuance of gastralgia deranges the health, and may even cause excessive emaciation, especially if the complaint be mistaken, or improperly treated. The complexion, however, preserves its freshness. And if, in a few instances, we perceive some febrile movement in the system, it is by no means a proof of phlogosis in the stomach, but may result entirely from a neuralgic source. Intermittent fevers themselves, in their simple form, have nothing inflammatory at bottom.

VI. In somewhat violent and prolonged cases of gastralgia the patients experience difficulty of breathing, palpitations of the heart, wandering pains, and peculiar sensations of coldness, especially in the arms, loins, and lower extremities. Their sleep is sometimes good, sometimes agitated, sometimes null; yet, in the mornings, they get up refreshed and feel quite well, till breakfast renews the gastric sensibility. Nothing of this kind obtains in latent gastritis.

VII. Those who are affected with chronic inflammation of the digestive tube, are melancholy, morose, and impatient—but this is nothing to the state of moral depression and anxiety which obtains in gastralgia. In this last, there is ineffable despondency—disgust of life or fear of death in the extreme—the slightest sensation in the stomach awakens the patient's terrors—he is tremblingly alive to every look of his physician—to every word which is spoken by his friends respecting his complaint. He is afraid of taking any thing into his stomach, and by this he aggravates the complaint—he is convinced that his disease is mortal—becomes entirely absorbed by his own sensations, and indifferent to every thing else. But any diminution or temporary cessation of the gastralgia immediately changes the scene from despair to sanguine hope—to be again reversed on the slightest accession of the pain. Thus he goes on alternately desponding and hoping, till health is ultimately established.

Can any one suppose that these two opposite trains of phenomena, as presented in gastralgia and gastritis, are dependent on the same pathological condition of the organ affected? Although reason and observation must convince most practitioners that these two states cannot be identical, yet it is not so easy to prove their diversity. If patients died of gastralgia, dissection would throw some light on the point in litigation; but they do not die unless the disease passes into gastritis, and then the disciples of the new doctrine consider themselves as confirmed in their former diagnosis. But it may be urged, and with more shew of reason, that gastralgia is the first stage of gastritis—and that *inflammation* must always be preceded by more or less of *irritation* or affection of the nervous system of the part. If we admitted this in a physiological or pathological point of view, we could make little use of it in practice—nay, it would be of positive disuse. The treatment of gastralgia differs, *toto cœlo*, from that of gastritis—and what is useful in the one disease would be injurious in the other. This we have so repeatedly witnessed, that we cannot draw too much the attention of the young practitioner to the distinction between the two affections, more especially as there is a prevailing mania, both in this country and on the Continent, to amalgamate the two states, under the term chronic inflammation of the mucous membrane of the stomach and alimentary canal. The diagnosis between these two diseases is greatly assisted by an examination of their etiology—a study of the utmost importance in all cases.

*Etiology.* I. The most common causes of *chronic gastro-enteritis* are:—The abuse of spirituous liquors—stimulating medicines—emetics (this does not apply to England, and is probably an exaggerated cause in all countries)—drastic purgatives—poisonous substances—the presence of foreign bodies in the alimentary passages—too stimulant diet—excesses at table—ices and cold drink taken when the body is heated—vicissitudes of temperature, especially from hot to cold—particular temperaments, as the sanguineous, which dispose to the phlegmasiæ—suppression of hæmorrhages or other accustomed evacuations—repercussion of cutaneous diseases—rheumatic and arthritic metastases—contusions on the regions of the stomach or bowels.

II. Let us take a view of the causes of gastralgic affections. These are; a nervous and irritable temperament—sedentary habits—desk-work, and mental exertion—all the passions, when in excess, as jealousy, ambition, envy, &c.—disappointments, losses, and vexations of mind—certain trades or professions, as tailoring for example—venereal excesses—hot and moist atmosphere—fear of having gastro-enteritis, among medical and literary patients—too lowering a diet for other complaints—long fasting—too much liquid and slops—excess in tea-drinking—in one word, every thing which can inordinately exalt, directly or indirectly, the nervous sensibility or susceptibility of the stomach. Much error, we are persuaded, has arisen from considering as similar, the effects arising from different morbid causes acting on the stomach. We will take two causes for example—the immoderate use of *ardent spirits*, and *profound grief or disappointment*. Gastralgia may and does often result from both these causes; but are we to infer that it is of the same nature in both cases? Certainly not. In the *former*, it is likely to be of an inflammatory, in the *latter*, of a purely *nervous* character. We grant that it may be difficult to ascertain, in the first instance, what is the real character of the gastralgia, as the causes are often studiously concealed from our view. But surely, in such cases, it is wise to be guided by the effects produced by medicinal agents, and not wildly rush on, from a preconceived theory. If we find that the pain in the stomach is increased by antiphlogistics and starvation, ought we not to make a cautious trial of the opposite plan—sedatives and tonics? But the disciples of Broussais (the gastromaniacs) deny that we can learn any thing of the nature of a disease from the effects of the medicines employed in the treatment—a very convenient disbelief certainly! We maintain, on the other hand, that the nature of a disease is often determined, and can often be determined *only*, by the effects of remedies. This is a truth which every practitioner knows well.

In this place our author makes a remark, the justice of which we can vouch for—and indeed we have more than once endeavoured to impress it on the minds of our brethren. In gastralgic and dyspeptic affections the patient is desired, as a routine rule, to eat *little and often*. This appears to have some support from the fact that the patient with gastralgia often feels a craving for food a few hours after having taken nourishment. But woe to him who has the imprudence to satisfy this craving! It is a false hunger which ought to be borne with patience. The stomach, like every other muscle, requires its periods of repose, and we are convinced that there are but few cases where the regular hours of eating should be interfered with. These may be more frequent where there have been great losses of blood, or exhaustion from some severe disease. In the convalescence from such accidents the stomach, in the name of the whole system, demands more frequent supplies of food than in a state of health, and digests it more rapidly. But these are very different from cases of gastralgia and dyspepsia.

In conclusion, our author admits that there are cases where gastralgia and gastritis are combined, and then the treatment becomes very difficult. The very combination itself is probably sometimes produced by a too stimulant regimen or medication for the cure of gastralgia. Irritation may and does pass sometimes into a state of inflammation—and the wonder is that it does not oftener change thus, on the principle "*ubi dolor, ibi affluxus.*" No rules of conduct can be laid down for the diagnosis or treatment of such combinations, and the practitioner must trust to his own sagacity and discretion in each particular case. We conceive that the present paper will do much good, especially on the continent, where gastro and enterito-mania is the order of the day.

"It is almost inconceivable," says our author, "with what readiness our physiological physicians admit the existence of gastric inflammation. If we are to believe them, the stomach is a kind of powder-magazine, which is exploded by the most trifling spark, and by means of the most opposite nature. Nay, it takes fire *spontaneously*, according to their account, like a stack of damp hay—and gastro-enteritis, acute or chronic, continued or intermittent, regular or irregular, sporadic or epidemic, contagious or non-contagious, usurps almost the whole nosological chart, under the denominations of plague, typhus, small-pox, measles, scarlatina, fevers of all types, cholera morbus, gastralgia, bulimia, hypochondriasis, &c. In short, it is a perfect Proteus, with all kinds of shapes at command—a complete Hydra, whose heads give employment to the whole faculty, and grow again as fast as they are lopped off." If for *gastro-enteritis* we substitute "derangements of the digestive organs," the satire will perfectly apply to the *gastro-mania* of this country. "Mutato nomine, de te fabula narratur."

---

2. SPASMODIC CONTRACTION OF MUSCULAR TUBES.

Dr. A. Monro has favoured the profession with a paper on this subject, in our contemporary of the north—the Edinburgh Journal of Medical Science, of which we shall take a short notice. We think the worthy Professor has wasted time in discussing the question of muscularity in certain tubes, as the urethra and bile ducts. Suppose the eye fails to detect *muscular* fibres in these tubes, will any one be hardy enough to deny that they possess *contractility?* There are very few tissues or structures in the body that are perfectly devoid of this quality—and that the tubes above-mentioned are endowed with it, and in no mean degree, every pathologist must have had ample opportunities of proving. Passing over fifteen or sixteen pages, therefore, of matter which is not not very interesting to the practitioner, we come to the pith of the communication.

1. *Spasm of the Gullet.* This is a far more frequent disease or rather disorder, than is imagined, especially in females. The spasm is oftener partial than general, the constriction occurring most commonly at the union of the gullet with the stomach. In such cases the patient feels as if some extraneous body were lodged within the œsophagus, accompanied by an ascent of air, the stricture preventing it going off by eructation. The food is either detained for some time, and then reaches the stomach, or is rejected as soon as it touches the spasmed part. The symptoms of spasm in the œsophagus, in addition to the foregoing, are, sudden loss of power of deglutition, accompanied by sense of constriction in the fauces, which have a parched appearance—great weakness and emaciation, if the disease continue. The seat of the spasm can only be ascertained by the probang, which will be firmly grasped by the contracted part of the tube. This spasm of the œsophagus sometimes lasts only an hour or two, at others for days, weeks, or years. Dr. M. relates some particulars of a curious case that occurred in the Royal Infirmary of Edinburgh, in 1792. The patient was a young woman who had long laboured under epilepsy, succeeded by paralysis of one of her limbs, and imperfect vision of one eye. She then gradually lost the power of swallowing, and at length upon attempting it, was seized with convulsive attacks. Soups and nutritious substances were introduced into the stomach by means of a tube, and by this process she was nourished for two years and eight months. She gradually recovered the power of swallowing, and enjoyed a tolerable share of health. Having afterwards died of inflammation of the lungs, the œsophagus, on dissection, was found free from disease.

Hoffman relates a remarkable case of a man who, in consequence of excessive grief, was seized with spasm of the pharynx and difficult deglutition, together with the sense of a foreign body thrust down his throat. In the accessions of spasmodic contraction he had shivering, constipation, formation of wind in the intestines, want of sleep, hard pulse, limpid urine. This disease lasted three months, with intervals between the paroxysms. The patient is said to have been cured by medicines prescribed by Hoffman—chiefly his own liquor anodynus; but we are disposed to think that Nature and time were the principal agents in the cure. This complaint is by no means uncommon among nervous and hysterical females. The treatment will be alluded to presently.

2. *Spasm of the Stomach.* This is one of the most painful complaints, while it lasts, that human nature is liable to. It comes on in paroxysms of acute pain which almost entirely stop the breath, attended with weak pulse, palpitation, and sometimes sudden death. Indeed it is quite impossible for life to continue very many minutes under severe spasm of the stomach. Fortunately it remits, and thus gives the unhappy patient a respite between the fits. It is generally produced by irritating or indigestible substances eaten as food, when the stomach and digestive organs are disordered. Gout is well known to attack the stomach in this form, and to prove a very dangerous disease.

3. *Spasm of the Intestines.* Colic is so common and familiar to the practitioner, that we hardly expected Dr. Monro would have gone through the ceremony of describing it, at this time of day. The Doctor gives us the titles of eleven kinds of colic, and moreover a list of the various authors who have written on the complaint, from

Alberti to Merat. This is all very erudite, and such as should usually appear from the Caledonian Athens. It is not, however, what the practitioner looks for in a paper in a periodical journal. Practical facts are there in their proper place and station. There are some species of colic which are readily distinguishable from others, as the colica pictonum, for instance; but there are varieties introduced by Dr. Monro which it would be somewhat difficult to discriminate at the bed-side of sickness, as the colica spastica, phlogistica, calculosa, scorbutica, hepatica, &c. These are very neat articles of furniture for a lecture-room, or an inaugural dissertation; but they are rather in the way of the practitioner employed in active scenes and occupations.

3. *Spasm of the Biliary Ducts.* Without denying the muscular structure of the bile ducts, Dr. Monro does not believe that spasm is so often a cause of jaundice, as has been stated by authors. Simple spasm, independent of any other morbid condition, must, we think, be a rather unusual cause of jaundice, though in sudden emotions of the mind, this would appear to be sometimes the case. But when the biliary secretion is depraved and viscid, obstructing the ducts, it is not improbable that spasm then comes on, in the same way as it takes place when gall-stones are impacted in the tubes. Gall-stones, says Dr. Monro, while passing through the cystic duct, create pain and sickness of the stomach, but do not prove a source of jaundice. This position we are inclined to doubt. The vicinity of a calculus in the cystic duct may readily obstruct the hepatic or the ductus communis, and thus produce jaundice, as may be easily conceived, and the history of cases confirms this. The symptoms of gall-stones impacted in, or passing the gall-ducts, which are stated by Dr. Monro, are those found in every book, from Thomas's Practice upwards.

4. *Spasm of the Urinary Bladder.* We look upon the following symptomatology of spasm in the bladder, as of very equivocal character. "The earlier symptoms of the disease are, a dull pain accompanied by a sense of constriction in the organ affected; and this pain extends along the ureters to the kidneys, loins, and thighs. An itching of the glans penis, and *frequent* erections are *not unfrequent.* The contracted bladder, by pressing on the rectum, impedes the exit of the fæces, which is followed by pain in the belly, and considerable distention of the bowels. Owing to the continued impediment to the passage of urine, the valvular communication of the ureters is forced, and the urine regurgitates and distends these canals, which is denoted by acute pain in the region of the kidneys. The patient has at the time, urinous sweats, and even his breath smells of urine. The spasm is sometimes succeeded by inflammation of the bladder." We acknowledge ourselves at a loss to know how all these phenomena were ascertained to be the consequence of *spasm* of the bladder. Dr. Monro cannot mean either the hour-glass contraction of the bladder, or spasm of the neck of that organ, as these affections are afterwards described, with their appropriate symptoms. He must therefore mean general spasm of the whole of the bladder, and if so we should be exceedingly obliged to the Doctor to favour his brethren with some of the cases on which the foregoing symptomatology is founded.

*Hour-glass Contraction.* When a catheter is passed up, in such a case, only a few ounces of urine are discharged, and a tumour can still be felt in the pubic region. We are informed that the late Dr. Clark, being aware of this circumstance, passed a gum-elastic catheter through the contracted portion, and thus drew off the urine from both the cavities of the bladder.

In respect to spasm of the neck of the bladder, with its accompaniment, retention of urine, we need say nothing. The symptoms are well known, and it was hardly worth Dr. Monro's while to record them again. The same remark applies to spasmodic stricture of the urethra, of which Dr. M. has given a very meagre account, occupying about a page.

*Treatment.* Dr. M. informs us that, in order to remove the spasmodic contraction of the passages for the food, the bile, and the urine, it is necessary to remove the cause, if possible. This is saying little more than that it is necessary to remove the disease. Thus, impacted fæces in the colon—a gall-stone in the biliary duct—a calculus in the bladder or urethra, will cause spasmodic contractions in those parts, and the removal of these is, of course, the removal of the disease. But to remove the causes we must often attend first to the effects. We agree with Dr. Monro that, "when much pain and irritation exist, the detraction of blood, and the tepid bath should precede the use of opium and other antispasmodic remedies." In respect to impacted gall-stones, we are sorry to say that we have less confidence in the power of medicine than most of our brethren. We are afraid that we can do little else than prevent mischief from inflammation in such cases, as we know of no medicine which has any power in propelling forward the concretion. The best way to relax the spasm when it exists, is by local depletion from the epigastrium, warm baths, and opium. We lately saw a rapidly fatal case of this kind in the person of a gentleman (Mr. S. of Lewisham) to whom we were called in the night. He had then laboured for six days under severe pain in the region of the gall-ducts, vomiting, headache, and latterly, fever. During this period not a trace of bile appeared in the motions, while the urine was like blood, and the skin, first yellow, and afterwards of a mahogany hue. We found him with delirium, black tongue, pulse 130, and the epigastrium so tender as not to bear the slightest pressure. The intelligent surgeon by whom he was attended, had used all the remedies which are recommended in such cases, but without any effect, and in 36 hours after this period the gentleman died. Dr. Beck saw him in this interval. Unfortunately we could not procure permission to examine the body; but there can be no doubt that a mechanical obstruction existed in the bile ducts, and that inflammation had supervened on spasm, by which these tubes were probably rendered impervious. The case approached pretty closely to what might be termed the green or black jaundice.—When fever and delirium arise in this disease, they are very formidable symptoms.

While writing these observations (July 7th, 1826) we had an opportunity of witnessing the happy effects of medical assistance promptly administered, in a case equally threatening, but where the obstruction was in the intestinal tube. A young man in the Strand, after eating imprudently of fruit and new potatoes, was seized with severe pain in the abdomen, between the umbilicus and left ilium, attended by vomiting, and complete constipation. Mr. Wilson, a very able surgeon, in the East India Company's service, who was acquainted with the family, bled this young man freely, administered purgatives, and threw up injections. These failing, the assistance of the writer of this article was requested. The young man was still writhing with pain, yet the skin was cool, the pulse natural, and the tongue clean. Under these circumstances we considered the disease, in its then stage, as impacted fæces attended by spasm. A large dose of calomel, with two grains of opium, were ordered, and after a couple of hours, purgatives and enemata to be administered. The pain was lulled, and some trifling evacuations procured, but next day the symptoms were all *exasperated*, and now the pulse began to rise, and the tongue to become furred. Leeches were applied to the abdomen, with hot fomentations; and purgative enemata were thrown up, while cathartics of active quality but small in form, were administered by the mouth. These means failing, we again opened a vein, and bled to syncope. Immediately after this, the bowels gave way, and immense quantities of fæces were discharged, with instant relief of all the symptoms. Here, then, was spasm from accumulations in the colon. Venesection, calomel, opium, cathartics, numerous enemata, leeches, and fomentations failed. But symptoms of inflammation at length supervened—and *then* the venesection had all the effect which could be desired. This case may not be incapable of affording some useful practical reflections, and we leave it to our brethren for this purpose.

In conclusion, we may observe that we were rather disappointed in the perusal of this paper of Dr. Monro's (occupying 32 pages) seeing the high authority whence it emanates, and the distinguished situation in which it is placed by our respected cotemporary of the North. But we hope for a more important communication from the same pen, in one of the succeeding numbers of the same journal.

---

### 3. ON PERICARDITIS.—BY M. LOUIS.

Laennec, after most accurately describing the pathological characters of pericarditis, acknowledges that he has sometimes been right in his diagnosis respecting it, but that he knows of no certain mark by which to recognize so serious a disease in the living body. Any contribution towards a more secure diagnosis must, therefore, be acceptable. Two cases of this disease, as carefully noted at La Charité, by M. Louis, are published in the January Number of La Revue Medicale, and of these we propose to give some account.

*Case* 1. A stone-mason, 27 years of age, of strong constitution, and who never had had any previous illness, was admitted into the Charité on the 11th August, 1825, having then been ill about a week, and confined to bed three days. The complaint had commenced, without any known cause, by acute pain in the left side of the chest, in the epigastrium, and between the shoulders, accompanied by dyspnœa, palpitations, and latterly by diarrhœa. The palpitations ceased on the third day, but the other symptoms continued, and cough was superadded. There had been no shiverings. Fifteen leeches had been applied to the epigastrium on the fourth day of his illness, but without any benefit. 12th, It was observed that there was some infiltration in the upper and lower extremities—face exhibiting some purplish tint—embarrassment in speech and muscular motion—great sense of debility—prominence without œdema, of the left side of the chest, but of a circumscribed kind—no respiratory murmur in this part, where pressure was painful, and percussion elicited no clear sound. The pulsations of the heart were not regular or distinct—pulse quick, small, feeble, and very irregular, as well as intermittent—jugular veins dilated—respiration rather quick—chest, except at the part abovementioned, sonorous on percussion. The patient did not complain of any particular affection, but only of general malaise. To be *bled to* ℥xij.—*lowest diet.* When six ounces were abstracted the patient fainted. In the evening twenty leeches were applied to the precordial region. 13th, No alteration. A blister to the salient part of the chest. 14th, Somewhat less oppression, and the pulse rather more regular. The progress of the malady was slow but sure. He was very closely watched from this time till the 25th October, the date of his death, a space of more than two months, in which time the following circumstances, among others were noted. On the 30th August an erysipelas was developed around the blister, and spread in stripes to different parts of the chest. About this time the dyspnœa was sensibly augmented—the patient was obliged to lie with his head high, and frequently started up in the night. On the 15th September, when examined by auscultation it was found that no sound could be heard in two-thirds of the right side of the chest inferiorly—the state of the left side remaining the same. On the 9th October no sound by percussion or stethoscope, could be heard in the inferior and posterior half of either of the sides. The dyspnœa was always considerable—the decubitus difficult, and the pulse irregular, with some cough and sanguineous expectoration. The lower extremities were also more and more infiltrated. He died in this state on the 25th October.

*Dissection.* We shall pass over the appearances in the head and some other parts, as not essential to the pathology of the case. On raising the sternum, nothing but the pericardium could be seen, it appearing to occupy the greater part of the chest. It contained a pint and a half of reddish serum—was rough and unequal on its internal surface, resembling an orange in colour, and lined with a false membrane, a line in thickness, firmly adherent. The surface of the heart resembled the rind of a pine-apple, and was also covered with a false membrane, in some places from two to five lines in thickness. The heart itself was rather smaller than natural, and its orifices perfectly sound. The aorta and pulmonary artery presented nothing remarkable.

In the above case there is a perfect correspondence between the symptoms and the *post-mortem* changes. The diagnosis cannot, in such cases, be difficult.

*Case* 2. An unmarried woman, 47 years of age, was admitted into LA CHARITÉ (under M. Chomel) on the 19th October, 1824. After experiencing violent mental anxiety, and having made a journey on foot, of more than 500 miles, which she performed in twenty days, she was seized, all at once, two hours after arriving in Paris, with a sense of terrible constriction and lancinating pains in the centre of the chest. To these symptoms was added palpitation, which went off in three or four hours, and did not return for three days. The other symptoms continued, the sense of oppression daily increasing, as also the difficulty of lying down in bed. She had no head-ache, giddiness, lipothymia, thirst, heat, or shiverings. The appetite was completely gone—motions regular. She had some cough. Leeches had been applied to the chest, without any good effect. She walked, though very slowly, to the hospital. *20th October*. This was the 26th day of the complaint. The countenance was sallow, and expressive of fatigue and malaise—decubitus difficilis—great oppression at the chest—pulse rather small, unequal, and irregular, varying from 68 to 80 in the minute—action of the heart unequal and tumultuous—chest sonorous throughout, except in the præcordial region, where the sound was dull for a considerable space, in which she complained of pain—occasional cough, with trifling expectoration—tongue white—anorexy—dreadful sense of tenderness when slightly pressed on the epigastrium—stools and urine natural. *Twenty leeches to the præcordial region—infusion of violets—lowest diet.* The following day presented no alteration; and from this time till the *1st November* (the day of her death) the symptoms above-mentioned gradually increased in intensity—the oppression became extreme—the sense of suffocation imminent, especially in the night, compelling her to keep constantly upright. In this dreadful state the poor woman retained the perfect possession of her intellectual faculties—and, ten minutes before her death, she told M. Chomel that the resources of his art were at an end. Such were her sufferings!

*Dissection.* The lower extremities were observed to be considerably infiltrated. The pericardium appeared much distended, and contained a pint of reddish fluid. The heart was of moderate size, but completely covered with a false membrane, which did not extend to the internal surface of the pericardium. There was nothing remarkable in the cavities, ostia, or great vessels of the heart. Some part of the right lung was slightly hepatised—the left, sound. There were some other morbid parts in the body, but evidently having nothing to do with the patient's death.

It is but rarely that we see examples like the above, of carditis or pericarditis, unaccompanied with inflammation of the pleura, lungs, or other organs, by which the symptoms are rendered more complicated. So far the cases detailed are valuable to those who wish to acquire accurate ideas of symptomatology. This last kind of knowledge is the most important of all. It is of every day application, and it is generally that which distinguishes the good from the bad physician. Symptomatology may do a great deal without the knowledge of morbid anatomy—as was seen among the ancients—but pathology cannot move a step in safety, without the assistance of symptomatology.

---

### 4. CASE OF DISTENDED BLADDER.

In the May number of the Repository is a case of great distension of the urinary bladder, by Mr. Thompson of Whitehaven, which, as it is rather rare, and certainly interesting, we shall notice here.

Mr. Baker had suffered long from complaint in his bowels, with progressive enlargement of the abdomen; the affection had come on insidiously, and his constitution had given way before it. *October* 11*th*, He was visited by Mr. Thompson to give an opinion as to the necessity of his being tapped. The symptoms were, dull pain and slight soreness on pressure, in the abdomen; a well defined swelling, reaching from the pubes to the cartilages of the ribs, having a sense of fluctuation; bowels irregular; motions generally white-coloured, at times streaked with blood; frequent desire to make water, passing, with difficulty, about a quart a day; low irritative fever; sleep disturbed; appetite gone; bilious hue, which, together with the pain in the abdomen, continued to increase. Conceiving this to be enlargement of the bladder, and apprehending scirrhus and enlarged prostate, Mr. T. directed the employment of the catheter, which, however, after repeated trials, he was told could not be introduced. An irritating application was applied to the abdomen, and a mixture of bals. peruv. tinct. digital. and vin. colchici given with anodyne mercurial pill at night. *February* 2*nd*, He died, and next day, in the presence of Messrs. Robinson, Fox, Wilkinson, and Thompson, the body was opened.

*Dissection.* On laying open the abdominal cavity, the peritoneum was found much thickened and very strongly attached to an oval body, which extended between the pubes and cartilages of the ribs filling the abdomen from side to side, and pressing up the intestines. This, when cleared of its diseased attachments and removed, was found to be the bladder, containing a gallon of urine, almost black; when quite full it held twelve pints. Its coats were much thickened; prostate gland greatly enlarged, pressing on and narrowing the urethra; anterior part of the rectum of a scirrhous hardness. All these parts were highly vascular. To the external part of the bladder and prostate were attached, by minute pedicles, innumerable small bodies of a spherical shape; no such appearance on the internal surface of the viscus.

Liver smaller and harder than usual; the gall-bladder was double the natural size, and contained a quantity of very thick bile; the cystic duct at its origin, was as large as a Spanish nut, and of very fine texture: from this to its junction with the hepatic, it was impervious: the calibre of the ductus communis was much diminished. Other cavities not examined.

*Remarks.* The necessity of attending to the state of the bladder, observes our intelligent author, in cases of supposed ascites, is to be borne in mind, particularly when we recollect that even John Hunter punctured this viscus, and only discovered his mistake by tasting the urine. With regard to the thickening of the muscular fibres, in cases of great distension of the urinary organ, it is evident that it is a remedial process, for were it not to happen the bladder must inevitably burst. As to the question whether it be from disorganization that the bladder does not resume its functions when the distension is removed, we should be inclined to think that it is not. We should rather think that this arose partly from the want of the accustomed *stimulus* to action, namely the quantity and pressure of the contained fluid, but principally from loss of tone. Mr. T. seems to imagine that the spherical bodies found on the outside of the bladder, were composed of the triple phosphates, formed from the exudation of the urine through its coats. We cannot conceive this to be the case. There may be inflammation and sloughing and so the urine may get extravasated; but we confess we cannot make up our minds to its exudation. What the bodies in question were, we cannot say; that they were not formed as is supposed, we think we can.

---

### 5. M. GUILBERT ON LEECHING THE OS UTERI.*

Some discussion arose in this country, not long since, on the merit and on the priority of applying leeches to internal surfaces of the body;—but it would appear that this operation, like many others supposed to be novel, can be traced back to a considerable distance in the records of medicine. In respect to the application of leeches to the os uteri, M. Guilbert has been at the pains of proving that the measure which he recommends has been noticed long before his own time, and consequently that he is not the discoverer. Thus Stahl, in his Memoir "*de Utilitate Sanguisugarum,*" has quoted from Zacutus Lusitanus a

* Considerations Pratiques sur certaines Affections de l'Uterus, en particulier sur la Phlegmasie Chronique, avec Engorgement du Col de cet Organe, et sur les Avantages de l'Application immediate des Sangsues dans cette Maladie. Par J. N. GUILBERT, M. D. Professeur de la Faculté de Médecine, &c. Paris, 8vo, 1826.

case where phrenitis took place from retention of the lochia, and where, all other means failing, four leeches were applied immediately to the uterus, and followed by a large discharge and complete relief. On examining the original work, M. Guilbert found that these were the words used :—“ *Quatuor hirudines filo appensas apponere gubeo, ea quarum suctu secuta longa evacuatione, &c.*” He thinks, and not without reason, that this application was merely to the internal surface of the vagina, and not to the os uteri.

Afterwards, however, a physician of Ferara, Jerome Negrisoli, repeated this operation, on a great number of occasions, and with great success, according to his own account. He published a small work on the subject, from which M. Guilbert has made some extracts. It is dated in 1665. In this case also, our author has pretty clearly shewn, the leeches were applied to the internal surface of the vagina, rather than to the orificium uteri. It is certain that, in the writings of Ambrose Paré, there is a passage, unequivocally expressing the cervix uteri as a part where leeches might be placed. “ *Pareillement l'application des sangsues au col de la matrice est utile.*” Paré says he quotes from Sylvius, but M. Guilbert could find no such passage in the writings of the latter.

But as we never deemed it of very great importance whether an operation be quite new, provided it be useful, we shall come at once to M. Guilbert himself.

Every practitioner, he observes, must have remarked the frequency of chronic inflammations in the uterus, with engorgement of the cervix of that organ, and also the general inefficacy of the means usually employed to remove those affections. “ I therefore,” says he, “ beg leave to propose a method of cure which I have found useful when all others had failed.” Our author read a paper on this subject five years ago, before a learned society, and the accumulation of facts has now induced him to make the remedy still more public, through the medium of the press.

He remarks that chronic phlegmasiæ of the uterus are apt to occur from various causes, especially after child-birth. These phlegmasiæ occupy sometimes the entire of the uterus, but more frequently the cervix. Acute inflammation of this part is, of course, readily recognized by the practitioner; but when *chronic*, it is often concealed, through false modesty, by the patient, and the medical attendant is not aware of the existing malady—till progress has been made to an irremediable scirrhus or cancer of the womb. Some patients complain only of a sense of heat in the parts—others of pains—or a feeling of weight—or bearing down, as if they had a prolapsus uteri, for which pessaries are sometimes prescribed, which do more harm than good. Some females with this complaint consider that they have the menses too abundant—others that they are affected with leuchorrhœa. Our author was called to a young lady who was merely supposed to have hysteria, but in whom he readily recognized phlogosis of the cervix uteri. By removing this affection the hysteria soon disappeared. The following case was among the first which led our author to the measure which is the subject of this paper.

*Case.* A young lady, whom M. Guilbert had attended previously to her marriage, for various complaints, chiefly of a nervous and erysipelatous character, was much harrassed during her first pregnancy, with attacks of circumscribed peritoneal inflammation about the lower part of the abdomen, which were relieved by local bleeding, fomentations, and the common means. At the time of her accouchement a new peritonitis was developed, and combated successfully by the same means; but the patient went out too soon after delivery, in a voiture, during cold weather, by which the inflammation was renewed, accompanied this time by marked metritis. The accoucheur (M. Gardien) was again called in, and prescribed the means formerly employed, with promptness and decision. The peritoneal inflammation was subdued, and that of the uterus much diminished; but, in spite of every measure, there continued an engorgement of the cervix uteri. M. Recamier was then called in consultation, and, on accurate examination, ascertained a phlogosed state of the posterior part of the os tincæ, which was prominent in the form of a small tumour that threatened to suppurate. Every means having been employed to dissipate this inflammation and engorgement, without success, M. Guilbert thought of the direct application of the leeches to the part inflamed, as a measure more likely to succeed than any other. M. Recamier agreed readily to the proposal, and a *speculum uteri* having been introduced, it was found quite easy to apply four leeches, through this instrument to the tumefied and inflamed part. Very little pain was experienced by the patient from the bites of the leeches; and M. Guilbert here remarks that the mucous membranes are much less sensible than the skin to this kind of irritation. The application was renewed some days afterwards, and though relief was obtained, the pains continued in the part affected. Ten leeches were now applied. By examination with the speculum, it was ascertained that the second leeching had much blanched the inflamed parts:—this third operation completely removed all pain, inflammation, engorgement, and tumour; so that, on exploration with the speculum, the parts appeared quite natural and healthy. The patient was thus completely cured in a very short space of time.

Since the period when this case was communicated to the Medical Society of Paris, our author has had many opportunities of reiterating the operation, and with much advantage.

M. Guilbert, however, judiciously advises that, before an application of this kind be made, the usual means should first be tried. General and local bleeding, with aperients, fomentations, and revulsives, should precede the direct application of leeches to the os uteri. Many cases, remarks, and extracts from older authors, are given by M. Guilbert, but we conceive that it is sufficient to point out this measure to our brethren, in the brief manner we have here adopted, leaving its merits and applicability to be determined by future experience. Judging from analogy, it is reasonable to suppose that the direct abstraction of blood from an internal organ would be more efficacious in arresting inflammation there, than when blood is drawn from vessels at a distance. There are but few, if any, internal organs to which leeches could be applied, except the uterus—and even here there must be some difficulty, notwithstanding the statements of M. Guilbert. The greatest difficulty, however, in this country, would be the repugnance to the operation on the part of the patient—a repugnance, for obvious reasons, much less surmountable here than on the Continent. Still, in hospital practice, and even on many occasions in private life, the measure would be submitted to, if deemed necessary—and we cannot but think that instances are not unfrequent where leeching on inflamed or otherwise diseased cervix or os uteri might be of essential service in alleviating, if not curing, some of those terrible and painful diseases to which woman is, unfortunately, too prone, and for which our art is too barren of resources.

We need say nothing of the speculum which M. Guilbert uses, as the instrument constructed by Mr. Weiss is superior to any which can be procured in France.

---

### 6. IMPERFORATE ANUS.*

Mr. Hutchison observes that, so little has been written on the subject of imperforate anus, and so few cases recorded, while, at the same time, the lusus is more frequent than is generally supposed, that he deems it a duty to the profession to lay before them such observations as have occurred to him on this point of surgery, in the course of his public and private practice. We acknowledge the duty, and praise the laudable desire to fulfil it; but we cannot acquiesce in the statement that few cases of imperforate anus are on record. The periodical Journals of this and of other countries contain numerous cases of this congenital defect, and even professed memoirs have been written on the subject, of which we need only instance the "*Memoire sur les Enfans qui Naissent sans un Veritable Anus,*" *par M. Bertin, 4to. Paris,* 1774. Hildanus, Scultetus, Petit, Van Swieten, and many others, have written, though not *ex professo*, on the same subject. We grant, however, that in the case which Mr. H. has particularly detailed, the extremity of the intestine was situated at a greater depth from the surface, than in any case of which we have read. To this then we shall immediately proceed, and afterwards make reference to some others scattered about in various works not very accessible to the generality

---

* Practical Observations in Surgery, &c. &c. (Second Edition considerably enlarged.) By A. COPLAND HUTCHISON, late Surgeon to the Royal Naval Hospital at Deal, &c. &c. &c. 8vo. 1826.

This new edition of Mr. Hutchison's work is much improved, as well as increased in the quantity of materials. We cannot review a second edition, but we can conscientiously state that every Naval and Military Surgeon, at least, should consider this volume as a necessary item in the list of his select and unavoidably restricted library. We have chosen the Chapter on "IMPERFORATE ANUS," for the present article of our Periscope, because it was not contained in the first edition, and because we are enabled to add some interesting particulars, from various other sources, to those presented by Mr. H. in the Chapter which he has dedicated to the subject in question.

of our readers. We may premise, however, that this was the fourth case which had occurred in Mr. Hutchison's practice, in the course of a few years. It is by far the most interesting of them all.

The child had been born 48 hours before the operation, which was performed in the presence of several medical gentlemen, on the 17th November, 1822. We shall give the operation in Mr. Hutchison's own words.

"The raphe was the only guide we had for the operation, there being neither hollow nor depression to mark the spot where Nature had failed in completing her design. Dr. Granville kindly assisted me (at the operation), by securing the child, and by keeping its lower extremities in a proper position during the operation, which was done by making an incision about an inch and a half in length with the scalpel, through the skin and fat, nearly as deep as the incision was long, but narrowing it two-thirds at its fundus. Not having reached the intestine with the scalpel, and considering that we could not so safely proceed further upwards in the direction of the gut with that instrument as with the trocar, the latter instrument was preferred, and directed gently upwards, backwards, and inclining to the direction of the sigmoid flexure of the colon for about an inch; when, on withdrawing the stilette, we found the intestine had not yet been reached: the stilette was therefore again passed through the canula, which was still kept in the parts, and pushed upwards half an inch farther, when, from a want of resistance, I suspected we had at length succeeded, and on withdrawing the stilette a second time, meconium flowed through the canula in considerable quantity; and here it was curious to witness the instinctive straining of the child to relieve itself for the first time, and which would suggest the advantage to be derived from the practice of gently irritating the external skin over the situation of the anus in such cases, with a view of ascertaining the probable distance of the gut fom the surface, as before noticed; for, on this occasion, the contorted features of the infant were precisely those of an adult who was constipated, and straining to relieve himself.

"The canula was secured by tapes, and retained in the parts three days. It was then withdrawn, cleaned, and again introduced, the fæces passing through it during that period." 267.

After a week or ten days the canula was removed, and a sponge-tent introduced; but did not expand enough. A bougie was substituted, and found to answer better. By this instrument the distance of the gut from the external surface was ascertained to be exactly three inches. Occasional constipation took place, and was obviated by castor oil. Two months had now elapsed since the operation, when the mother was directed to introduce the bougie for a few hours only every day, and then Mr. H. took his leave. At the end of three months the mother brought the child to Mr. H. reporting that she had that morning observed, for the first time, that the urine was tinged with fæces. Yet the child fed well and throve, till within six days of its death, which took place ten months after the operation.

*On dissection*, it was observed that the small intestines were all apparently on the left side, resting on the sigmoid flexure of the colon. The rectum was very large and distended with air. The lower part of this gut adhered to the bladder. The sphincter ani muscle was wanting. There was discovered a small valvular opening between the bladder and urethra, admitting only of the passage of thin fæces. The coats of the rectum were thickened, the muscular fibres being probably increased from the force required to project the fæces through the narrow canal leading from the termination of the gut to the external surface—a distance now of only one inch and a quarter. Mr. H. thinks, and with reason, that the lower part of the rectum had been, by its own muscular action, forced gradually down towards the external part, in the acts of stercoration, thus accounting for the shortening of the artificial canal since *the operation*. The substance through which the artificial anus passed was almost semicartilaginous.

This operation does great credit to Mr. Hutchison, though we are disposed to think that death was no great misfortune in the case of this child. Had the artificial canal still continued tight, and the parts through which it passed indurated, the patient's life must have been rendered miserable, and the gut above would have become greatly enlarged or diseased. On the other hand, had the artificial canal continued to shorten till at length the termination of the gut came close down to the external surface, the want of a sphincter ani would have been a terrible affliction, and rendered life a loathsome boon! Under whatever aspect then we view the case we believe that death was the best thing that could have happened.*

Since the foregoing case occurred, Mr. H. was called to another at Sheerness, where the gut was found completely closed about half an inch from the external opening. Mr. H. made an incision with the scalpel, of a proper length and depth, and then introduced a small-sized trocar, fully three inches; but no meconium passed. The patient died in a few hours afterwards, and it was found that the gut had been grazed but not penetrated. The rectum was found large and muscular, and pretty well filled. At the sigmoid flexure it passed immediately across and before the small bowels, "the upper part of the rectum rested on the cæcum, and then, turning from this situation, it passed down behind the bladder, and in close contact with it, and terminated in a cul de sac, only *one quarter of an inch* from the fundus of the *external cul de sac*, which arrested the progress of the bougie and probe." This was the precise state and position of the rectum in the other case already detailed. Mr. H. thinks it reasonable that this peculiar arrangement obtains in the majority of cases of imperforate anus, and that the practical conclusion to be drawn is, "that we must, in future, make

---

* Petit observes that where an operation is performed, and vent given to the fæces, if the sphincter be wanting, an artificial anus will be the consequence—"ce qui est un mal plus facheux que la mort n'est à cet age." In this sentiment we entirely coincide.—*Ed.*

our external incision, and point the trocar, when that instrument is necessary, more forwards in the direction of the bladder, than as before described in this chapter." We conceive it to be dangerous to draw any conclusion of this kind from two cases, however coincident they may appear, on the points abovementioned.

Mr. Hutchison makes many judicious remarks on the lusus naturæ under consideration, several of which, however, have been made by preceding writers. He thinks the operation should be deferred till from 24 to 60 hours after birth, as no great inconvenience can arise within this period, and it is necessary that the rectum should be somewhat distended with meconium when the operation is performed, otherwise the instrument coming in contact with an empty gut, might divide two or three of its folds, and thereby occasion mischief. He thinks the distance of the gut from the exterior surface may be pretty nearly estimated by certain external appearances. Thus, where the usual hollow between the nates exists, and the natural situation of the anus is well marked by a depression, "the intestine will be in proportion near; and where these do not exist at all, the chances are that it is at some distance." He does not lay this down, however, as an infallibie rule.

We shall now advert to the observations of some preceding writers on this imperfection. Bertin divides the lusus into four species, namely, 1st. Where the anus is obstructed by either a thin membrane or a more fleshy substance—2nd. Where the rectum is wanting, is not perforated through its whole extent, or terminates in one or two cul de sacs before reaching the external surface—3tio. Where the rectum terminates in the vagina, or bladder, or both, of the female.—4to. Where the gut terminates in the bladder of the male, or partly in the bladder, and partly in the usual place. A later continental writer makes seven species or varieties of imperforate anus, but they may almost all be comprehended in the above classification. In the first species, the infant's life may be saved, and a cure effected, if the aperture is made in time. If not, death generally takes place about the fifth or sixth day after birth. Nevertheless Hildanus performed the operation on the sixth day, with complete success. Ferrein saw an instance of life being protracted till the twelfth day, where there was entire imperforation of the anus. There is a species occasionally met with, where there is a very narrow passage in the natural situation, which will only permit the exit of fæces in a liquid state. Scultetus mentions a case where a stilet could hardly be introduced into the opening. The parents would not consent to an enlargement by the knife; but the object was obtained by tents.

That species where the occlusion is caused by a septum at some distance from the external orifice is the more dangerous, as it is sometimes undiscovered till too late. We may always suspect such a thing when laxatives fail to procure motions in the first day or two, and the infant appears in pain, and frequently straining. Many cases of this kind are on record.

There have been instances where this septum contained an aperture in its centre, sufficient to give issue to liquid or soft fæces. A man was received into the Hôtel Dieu in the year 1816, who had experienced great difficulty, from his birth, in voiding his motions, which were always, when not liquid, moulded into a cylinder of very slender dimensions. He had a stercoraceous fistula in perineo. On examination with the finger, a septum was discovered crossing the rectum, with a hole exactly in its centre, that would just admit the point of the finger. M. Dupuytren immediately enlarged this opening by means of a bistoury. In thirty-eight days this man was discharged cured of both complaints. When the cul de sac is at a considerable distance upwards, a trocar must be used; but it has unfortunately happened that the opening made into the gut in this way, when in a state of distention from the meconium, did not correspond with the other part of the artificial passage, after the gut had been emptied—the consequence was, a fæcal extravasation and death. This happened in the case of a child operated on by Sabatier, who died on the second day. A similar case occurred to Engeraud.

In cases similar to that detailed by Mr. Hutchison, some have succeeded, and others have failed after an operation. Saviard (obs. 5, p. 9) relates an instance where an infant was brought to him, without any external appearance of anus. He pushed in an abscess lancet in the direction of the gut, to the depth of three finger-breadths, when meconium issued along the side of the instrument. A tent was then introduced, and a passage effected for the fæces, with perfect success. An infant was lately brought to one of the most eminent surgeons of Paris, where no anus existed. An incision to the depth of two inches was made, but no rectum could be found. Next day the incision was carried deeper, but without success; and the child died. Here the trocar *ought* to have been used, as in Mr. Hutchison's case, and a farther chance of life given to the little sufferer. But even when the gut is reached by knife or trocar, and the meconium discharged, success does not always follow. J. L. Petit operated on an infant three days after birth, where no appearance of anus existed. The meconium was evacuated, but the child died in convulsions soon afterwards. In another case where he operated, he failed to reach the gut; but, three hours afterwards, a dark-coloured protrusion appeared in the wound, which was opened and gave vent to a quantity of meconium. The child was relieved, and lived till the eighth day, and then died. It was found on dissection, that the protrusion into the wound was a kind of hernia formed by a portion of gut pushed down during the violent strainings of the child. All the parts in the pelvis were in a state of gangrene. The same surgeon (Petit) operated on another child, first with the knife, and then with the trocar. The gut was perforated, and issue given to the fæcal matters, but death ensued on the second day.

Callisen, in his system of surgery, cautions us, when we find it necessary to use the trocar, to first empty the bladder by a catheter, which should be kept in the urethra as a guide during the operation. This eminent surgeon carried the incision in one case, to the depth of two inches, and succeeded in saving the life of the child. "Ad duos pollices, cum successu induximus, &c."

It is more than one hundred years since Littre proposed, in cases where an operation failed to reach the gut, to make an opening into the abdomen, in the iliac region, and establish an artificial anus in the colon. Strange and chimerical as this proposal may appear, it has actually been put into execution, and that too, with success, by M. Duret, surgeon to the Naval Hospital at Brest.* A child born without anus was presented to this surgeon. In the presence of several medical gentlemen he plunged a bistoury into the situation of the anus, and search was made for the gut, but without success. The child appeared doomed to death. The abdomen was distended, there was constant vomiting, and the extremities were getting cold. An incision was made over the sigmoid flexure of the colon, the gut secured to the edges of the wound by a silk thread, and an opening made into it. A large quantity of meconium issued. The symptoms were immediately mitigated, and the little infant recovered. It was seen twelve years afterwards at Brest, by M. Lassus, with an artificial anus in the situation above-mentioned. M. Pillor, of Rouen, has had similar success in a similar case.

There may doubtless exist a combination of *moral* circumstances which may render the preservation of life such a desirable object as to authorise the operation in question. But we much doubt whether the individual so rescued from death would have just cause to thank his deliverers in after-life. On this subject, however, we shall not dwell any longer.

When the rectum terminates in the bladder, it is almost always mortal, especially in boys. Yet Flagani reports a case where a child lived several months with this terrible lusus. The infant, when first seen by Flagani,† was four months old. It presented no trace of anus, and passed all fæcal matters by the urethra. Yet the child appeared hearty and well. Sometimes the abdomen swelled very much, and the infant seemed to suffer. Three months more passed on in this way, when it was determined to try the effect of an operation. A trocar was, therefore, pushed into the usual site of the rectum, but only some drops of blood followed. The instrument was then plunged in still deeper, but with no better success. The child lived a month after this operation, being harrassed with great pain and suffering every time that fæcal matters were attempted to be passed *per urethram.* On dissection, the intestines were found greatly distended, and filled with excrementitious matters. The rectum was three inches in length, and terminated in a narrow canal, four inches long, which, passing under the prostate gland, opened into the membranous part of the urethra. At this junction of the two organs was found a small cherry-stone, which the child had swallowed, and which had blocked up the passage for the fæces into the

* Recueil Periodique de la Societé de Médecine de Paris, tom. iv. p. 45.

† Osservazioni di Chirurgia, tom. iv, Obs. 39.

urethra. Morgagni, Bonetus, Desault and others have related examples of this kind of congenital defect.

When the rectum communicates with the vagina, as it sometimes does, and yet has an external orifice, attempts may be made to close the unnatural passage, by applying caustic, plugging the vagina, and enlarging the natural opening of the rectum by bougies. An instance is on record where a girl lived to the age of eight years, without any anus, the fæces passing all that time per vaginam. In these cases an operation, such as that practised by Mr. Hutchison, would bid fair to be successful, since the gut could not be at any great distance from the external surface. In such a case, a bent probe or other instrument could be passed into the communicating aperture, and the point of it pressed outwards to direct the incision.

Many curious cases of imperforate anus will be found in the second volume of Chirurgical Pathology, by M. Lassus, to which we refer the reader for further particulars.—In the English periodical journals are also scattered a considerable number of cases of this congenital defect.

We must now take leave of the subject, again repeating our conviction that Mr. Hutchison's volume contains a great deal of interesting matter, which the army and navy surgeon will do well to study, and refer to from time to time.

---

### 7. KIRRONOSIS.

A new medical journal, which promises to be of a superior character, is just established by some of the first medical men of Paris. The work is in quarto, and the first number contains about 100 pages. It is intended to come out every three months, under the name of *Répertoire Générale d'Anatomie et de Physiologie Pathologique, et de Clinique Chirurgicale, &c.*

Amongst the articles in the first number, is a short paper by J. F. Lobstein, of Strasbourg, on kirronosis. By this name the author distinguishes a remarkable affection of the fœtus or embryo, in which the serous and transparent membranes acquire a bright yellow colour. It is an internal jaundice, affecting the arachnoid, the pleura, the pericardium, and the peritoneum, but leaving untouched the ordinary seats of icterus, as the skin and the cellular tissue. It was first noticed in an embryo of five months. The peritoneum, especially where lining the posterior part of the abdominal parietes, was of an intensely yellow colour—that investing the intestines was less coloured. There was no effusion into the cavity, and the viscera themselves were healthy. In a second embryo of the same age, the abdomen presented a similar appearance, but in this instance the yellow colour also existed in the pleura, the pericardium, the arachnoid and dura mater, and in the thyroid gland. Lobstein has subsequently not only met with the same affection in the human fœtus, but has also had occasion to observe it in two embryo foals. A more careful examination shewed that the brain, the spinal cord, and also the nerves, participated in the morbid appearance. The sympathetic nerves, in particular, were very conspicuous, in the form of yellow lines passing by the sides of the spinal column. The affected parts appeared to be somewhat swollen, being larger than the portions immediately above and below them. The colouring principle in kirronosis does not seem to depend on any infiltration, but to be inherent in the tissues themselves. No adventitious matter could be detected by the aid of the microscope, yet the medullary matter of the spinal cord appeared to consist of two substances, the one of a bright citron colour, in the form of very minute grains, imbedded in the other, which was white and semi-transparent, and of a pulpy consistence. Long maceration in water, or alkohol, produces no change in the colouring principle. In preparations which have been in spirit seventeen years, it remains perfectly fresh, except in those spots which have been directly exposed to the light, which are a little faded.

Professor Lobstein is undecided as to the cause of this singular appearance. Having seen it as early as the third month, he cannot attribute it to the bile, which is not secreted at that period.

Kirronosis must not be confounded with an icteritious affection, sometimes met with in the fœtus, when produced at the full time, in which the cellular membrane is infiltrated with a yellow fluid. Lobstein relates a curious case of this kind, which co-existed with a complete disorganization of the cerebellum, the exterior of which was of a deep red, and formed a cyst of about the twelfth of an inch in thickness, containing a yellow albuminous matter, compared to an egg, after the yolk and white have been intimately mixed.

### 8. EXTRA-UTERINE PREGNANCY.

In the same number is an article by Breschet, on a particular form of extra-uterine pregnancy, which the author considers as new.* He remarks that three forms of extra-uterine pregnancy are at present admitted, viz. 1st. Graviditas abdominalis. 2nd. Graviditas tubaria, and 3rd. Graviditas ovaria. To these he proposes to add, 4th. Graviditas in substantia uteri, or graviditas interstitialis. He forms this new division on the authority of seven cases, of which he has published the description, accompanied with plates of four of them.

The first case occurred in June, 1823, in the practice of M. Belleman, physician to one of the dispensaries in Paris. The patient, who considered herself to be three months advanced in pregnancy complained of great pain in the hypogastric region, and extending to the rectum. Her

* This form he has described in the 13th volume of the Medico-Chirurgical Transactions; but, as we did not analyze his paper at the time, we shall take some notice of this species of extra-uterine pregnancy in this place, together with the commentaries of M. Geoffroy St. Hilaire, who was appointed by the Academy of Sciences to report upon it.—*Ed.*

countenance was pale, and her pulse small. She could retain nothing on her stomach, and had frequent syncope. These symptoms, which were attributed to peritonitis, resisted the means employed to relieve them, and the patient expired the following day. On inspection, a considerable quantity of blood was found diffused in the pelvis. The uterus, which was considerably enlarged, presented at its fundus and a little to the left, a rupture, in which the peritoneal coat participated. This opening gave exit to a fœtus enveloped in its membranes, but did not communicate with the cavity of the uterus, which, as in other cases of extra-uterine pregnancy, contained traces of a decidua. The parietes of the organ were upwards of an inch in thickness. The cavity which had enclosed the fœtus, was situated in its substance. It was about the size of a small egg, and was not lined by membrane. Its walls were thickest towards the uterine cavity, but even on the outer side they were derived from the structure of the uterus itself, as well as from the peritoneum. A fact decidedly opposed to those authors, who suppose that in cases of this kind, the ovum has slipped between the peritoneum and the uterus. There was no regular placenta, but the vessels of the chorion formed cotyledons, of which the filaments were implanted in the uterus. The ovaria were healthy. The right fallopian tube was half obliterated, and the left completely so.

The second example fell under the notice of the late Dr. Albers, who appears to have had the intention of sending to the Medico-Chirurgical Society of London, a description of the case, together with that of another, of which he had purchased the preparation. His death prevented the execution of this design.

Two other cases stand on record, the one in the first vol. of the Academy Josephine of Vienna, the other in the Archives of Horn. Two more which the author of the paper has related, appear to have occurred in the hospitals of Paris. The description of the first is taken from one of the Medical Journals, and its value is somewhat impaired by the author of the article having apparently misunderstood the case. The second is minutely detailed by M. Dance, who attended the patient in the Hôtel Dieu.

These cases all proved fatal from rupture of the uterus, and, with but one exception, this event took place at an early period of gestation. In all, the cavity containing the fœtus was situated near to the extremity of one of the fallopian tubes, which was invariably obliterated in at least a part of its course. In some there was a regular placenta, in others it was divided into cotyledons. The decidua was formed in the cavity of the uterus, and although the embryo had not descended into it, its dimensions were increased, and its parietes thickened. The author suggests various modes of accounting for this *graviditas in uteri substantiâ*, but whilst he admits that they are not all equally plausible, he refrains from so far committing himself, as decidedly to adopt any one of them.

The memoir from which the preceding extracts are taken, was read to the Academy of Sciences, and Geoffroy St. Hilaire having been appointed to revise it, brought in a report, in which he states his own views of the subject. Calling in to his assistance the doctrine of analogies, to which he has long devoted so much attention, he finds no difficulty in arriving at an explanation, which appears to be perfectly satisfactory to himself. Now, though we have no hesitation in expressing our conviction, that the theory of analogies, by affording the means of combining detached and scattered anatomical and physiological facts, renders a most important service to this branch of science, we are also aware that it opens a wide door to the imagination, and feel bound to hesitate before we admit the explanations which it offers.

In a future number, we may possibly have occasion to offer some remarks on the specious doctrine of the unity of formation. For the present we shall content ourselves with giving a short sketch of Geoffroy St. Hilaire's views of the cases before us.

He is not only opposed to the admission of a fourth variety of extra-uterine pregnancy, but thinks that the cases before noticed, as well as the three varieties acknowledged by the authors, who had previously treated of these subjects, are to be referred to one head. Judging, as it would seem, of the human embryo, by what happens with that of oviparous animals, he does not allow that any thing like fecundation, or development of an embryo, can take place, without the ovulum, which corresponds to the yolk, having previously acquired its different concentric coats of albuminous matter, and assumed the state of ovum, for which purpose it must first have traversed the fallopian tubes, and arrived in the corresponding turn of the uterus, or the ad uterum, the name which he gives to that part, which in the human he regards as analogous to the cornu of the uterus. In the fœtus traces of the uterine turns may often be distinctly made out, so as to indicate the analogy between the human uterus, and that of the inferior mammalia, and cases are not wanting in which this resemblance has become permanent, and been found in the adult.

Geoffroy St. Hilaire has taken advantage of these facts to support him in distinguishing as a special organ, the *ad uterum*, or that portion of the fallopian tube next to the uterus. He speaks of it as a part *sui generis*, constituting an essential appendage to the uterine system. He thinks the cases collected by Breschet are attributable to this part having either retained its fœtal development, or reproduced it under the influence of gestation, and thus afforded a receptacle, in which the ovum continued its growth, whilst the passage designed for it has become obliterated. He explains the cases of reputed *graviditas tubaria*, by supposing a retrograde movement of the ovum, when it has attained too large a size to remain in the ad uterum, and is shut out from the uterus. He states that the ovum may return to the surface of the ovary though no development of a vesicle can take place whilst it remains in that organ. It appears to us that the love of analogies has induced the learned Professor to attach too much importance to the accessions which the human ovum may receive, whilst in transitu from the ovary to the uterus.

The ova of birds in their passage through the oviducts are by various additions, prepared for being placed in those conditions, which are essential to the development of the embryos which they contain. Cut off, during the whole period of incubation, from every supply of nutriment, except that within themselves, it is needful that the store of this should be considerable, hence the large size of the yolks, compared with the ovula of the mammalia. The vital process cannot be maintained, through the indirect influence of the atmosphere on the blood of the parent. These ova must, therefore, be adapted to receive its more immediate influence, which they do through the porous texture of their envelopes, but as this arrangement allows of considerable loss by evaporation, ample provision is made against it in the watery part of the albuminous coverings which are superadded in the oviduct. In the lower part of this canal are also added the shells, by which the tender embryos with their appendages are protected from accidents and risks, to which the young of the mammalia, at a corresponding period of their existence, are not exposed. Even when the young bird has quitted the shell it still derives a part of its sustenance from the animalized matter which was supplied by its parent, in the form of yolk, and which in part remaining after the period of incubation has been completed, is taken up into the abdomen of the chick.

When we reflect on these essential differences between the development of the embryo in birds, and in the mammalia, and consider that Nature never gives herself needless trouble, we cannot but doubt the propriety of forcing an analogy between the passage of the egg, in the one case, through the oviduct, and in the other, through the fallopian tube to the uterus.

We do not deny that the human ovum in its course to the uterus may acquire some addition from the secretion of the mucous lining of the fallopian tube, but we suspect that it is of comparatively little importance, and confess that we are still inclined to retain the old opinion respecting extra-uterine pregnancies, rather than admit a retrograde movement of the ovum, especially when it has had time to attain considerable increase of size, as in the cases supposed by Geoffroy St. Hilaire. To say nothing of the restraint opposed to such movement, by the attachments of the placenta, or cotyledons, it would seem incredible that when the ovum can no longer be contained in that part of the tube which has been gradually dilated under its progressive growth, it should be able to slip through the unaltered part of that tube, which was only destined to give it passage in its earliest stage.

The fact that the traces of analogous structure in this part, which are observable in the fœtus, are, in most cases quickly obliterated, militates strongly against the importance which Geoffroy attributes to the ad uterum. Had it been well founded, the form of extra-uterine pregnancy noticed by Breschet, instead of being one of extreme rarity, ought, on the contrary, from the predisposition of the parts, to have been of frequent occurrence. We borrow another objection to Geoffroy's explanation, from the reasoning which he has himself employed in the present report. When, as in the case before us, we are examining a product of organization, at a period remote from its first formation, we are liable to see a sum of effects, unexplained by the actual state of the various parts, because dependent on the series of conditions which have gone before. There has been a continuous chain of transmutations, A producing B, and B producing C, and so on, until the final result may exhibit no traces of the type which formed its basis. Hence it is that analogies of structure in different animals, are most conspicuous in the first periods of their existence, the specific and individual peculiarities being produced and confirmed in the subsequent development. When the uterus becomes the subject of increased nutrition, in correspondence with the development of an embryo, we recognise the influence of a special cause, the effects of which may be modified by accidental circumstances, but cannot philosophically be supposed to be complicated by the operation of another cause, whose influence, as exhibited in the production of analogies, belongs to a totally different period of the individual's existence.

If we were to hazard an opinion respecting the cases of extra-uterine pregnancy, which form the subject of the two papers we have been examining, it would be that the passage of the ovum through the fallopian tube, a process known to occupy several hours, had not been effected before the formation of the decidua had closed the openings into the uterine cavity. The ovum having descended as far as this obstruction would permit, and formed those connexions which are necessary for its development, would dilate that part of the tube in which it was placed, at the expense of the adjacent parts. It is perhaps not impossible that, by this means, some of the fibres, which are most distinct at this part of the uterus, and constitute the *musculus orbicularis Ruyschii*, may be separated. The cavity containing the ovum would then be distended most readily in this direction, till at length the embryo would appear to be lodged in the substance of the uterus.

---

### 9. EXTRACT OF GARDEN LETTUCE.

The extract of garden lettuce, the lactucarium of the Edinburgh Pharmacopœia, has already been long known in this country, as affording, in some cases, a valuable substitute for opium. Dr. François, who has chosen to give to this substance the name of *thridace*, has just published some observations which tend to support the favourable opinion entertained respecting it, by some of our northern medical brethren. Dr. F. ascribes to it the power of allaying pain, of producing sleep, of diminishing the too frequent action of the heart, and of repressing inordinate heat, without producing the unpleasant effects of opium. He also praises it as an anti-phrodisiac. The dose which he gives, is from eight to ten grains.

Another plant of the same family, the lactuca virosa, has found an advocate in Dr. Thorle, who has employed it with success in hydrothorax, but as he has given it in conjunction with digitalis, it is not easy to determine how much of his praise really belongs to the lactuca.

10. DR. LOUIS' DIAGNOSIS OF PERICARDITIS.*

Dr. Louis, from the observation of thirty two cases of acute pericarditis, has been led to the opinion, that this affection, which Laennec and most other pathologists have regarded as one which presents the greatest difficulty in its diagnosis, may, when free from complication, be detected with almost the same facility as the plainest case of pleuritis. He offers the following as the symptoms which characterise it.

Pain in the region of the heart, sometimes extending to the back and epigastrium, very sudden in its attack, and in many cases, though not constantly, attended with palpitations. Irregularity and intermission of the pulse, and a dull sound yielded by that part of the chest which corresponds to the situation of the heart, whilst it is elsewhere naturally resonant. These symptoms are sometimes accompanied with syncope, and occasionally with œdema of the extremities. Dr. Louis lays particular stress on the absence of sound in the region of the heart. Although we are disposed to pay great respect to the opinion of one who stands so high as Dr. Louis deservedly does, as a patient and accurate observer of disease, yet, we cannot help thinking that pericarditis may frequently exist when this symptom is wanting. When present, it must depend on fluid in the pericardium, and, as this effusion is the effect, rather than the cause of the disease, it is reasonable to conclude, that the early and most important stage, must be passed before the symptom in question can be unequivocally developed. In some cases, when the inflammation has been very considerable, the quantity of fluid found on examination, has been very trifling, and instances are not wanting, although they must be regarded as rare, in which the pericardium, from a suspension of its proper secretion, has been seen morbidly dry. Another symptom said to indicate the existence of pericarditis, is a peculiar sound accompanying the contractions of the heart. It has been particularly noticed by Dr. Collin, who describes it as resembling the noise produced by the bending of new leather.

In a case of acute pericarditis, which, not long since, fell under our notice, the natural sound of the contractions of the heart was considerably perverted, though not so as to suggest the simile offered by Dr. Collin. It is highly probable that, in most instances, the sound may be modified, but the varieties in the quality, as well as the extent of the inflammatory products, are likely to subject it to almost infinite variations.

11. ON THE FREQUENT OCCURRENCE OF THE LOSS OF THE NOSE IN RUSSIA.

On a hasty glance at those authors who have written on the general

* This may be considered as a sequel to No. 3, p. 504, of this Periscope. —*Rev.*

state of health in Russia, one is immediately struck with the frequent occurrence of the loss of the nose. VON BESSARABSKEY, who has now a work in the press on this subject, sent a part of the manuscript copy to Dr. Edward Graefe, of Berlin, which, with the permission of the author, was inserted in the *Journal für Chirurgie*, from which we extract the following passage.

"Although the venereal disease has for some time past very much diminished in the Ukraine, although it is almost entirely banished from the peasantry, and only prevails in the towns, among the country nobility and the Israelites, yet its ravages are sufficiently important to merit a few observations. The raging, irrational, and almost brutal lasciviousness, with which the Polish Jews are upbraided, which may partly arise from their stimulating high-seasoned diet, is assigned as the reason why this horrible disease is found in its most varied forms, and with such frequency among that people. Syphilis is there extraordinarily obstinate and destructive; venereal ulcers extend themselves over the whole body, even over the face. As Storch very justly remarked in the last century, there is perhaps no country in Europe in which venereal ulcers of the nose so frequently occur, and no land in which one observes so many people without noses as in Poland. In Siberia, the loss of the nose is found equally frequent, indeed much more frequent, even than in Volhynia and Podolia. That the low temperature of that region may be partially the cause is very probable, but that it must also happen from mismanagement of the syphilitic inflammation attacking this part is equally so; as it is found that at Petersburg, where the winter is not at all milder, such cases are not near so frequent. As yet, as far as we know, no attempt has been made systematically to examine into the subject, and it is very desirable that such an examination should be made."

GMELIN, who made a tour through Siberia in 1750, observes, that he never saw so many people without noses as in the neighbourhood of Tobolski; and he found that the venereal disease was sadly mistreated among the people.* Out of 100 recruits brought out of the Ukraine in the latter part of the last century, 80 were found to have syphilis. Baron Bessarabskey's forthcoming work will be entitled "Nachrichten über Podolien, Bessarabien und die Bukowinan;" a work from which we expect much interesting information.

---

### 12. VAGITUS UTERINUS.

A lady, about thirty-four years of age, was, in April, 1824, delivered of a fine grown child. The pains, which at first set in very strong, became in the course of twenty-four hours very ineffectual; the intervals between the pains were long, and the labour made little or no progress for two days. The principal accoucheur of the neighbourhood was called, who almost immediately used the forceps to bring

* See *Gmelin's Reise* durch Siberien, Gottingen, 1751.

down the head of the child, which had a natural presentation. After the first blade had been introduced, the position of the head was altered a little, and the child gave, at that instant, although quite *within the uterus a distinct cry*, audible not only to the operator, but also to all the persons standing by. In a quarter of an hour after the child was delivered.—*Rust's Magazin für die gesammte Heilkunde*, 1825, XIX. *Band*, 2*tes. Heft.*

---

13. PERIODICAL APHONIA.

A young woman pregnant for the second time had, from the fifth month of her pregnancy, been frequently the subject of a temporary loss of voice. Preparatory to such aphonia, the patient complained of a weight and uneasiness similar to that produced by the aura epileptica, and, after an hour, somewhat like an aura proceeded from the feet to the neck, and the loss of speech followed; the patient however preserved the most undisturbed self-possession and consciousness. The countenance, during the continuance of the aphony, preserved its regular appearance; no spasm of the muscles or any other disturbance—pronunciation was alone prevented. The circulation, the respiration, and all the other functions were perfectly regular, &c. At each attack, a vein was opened in the foot, from which the patient invariably obtained relief, and she could tell by a peculiar feeling when blood enough had escaped. She had, namely, a sensation as if a heavy weight pressed upon her breast, scarcely was the bandage laid on before she felt better, and the power of speaking almost immediately returned. An attack of this kind returned several times during the gestation at an interval of fourteen days, and was always removed by the same treatment. The patient was eventually delivered of a full grown healthy child, since which time she has had no return of the affection, nor any epileptical or spasmodic symptoms.—*Rust's Magazin für die gesammte Heilkunde*, 1825.

---

14. HISTORY OF A PREGNANCY, WHICH ENDED IN THE DISCHARGE OF THE FŒTUS THROUGH AN ABSCESS IN THE UMBILICAL REGION, WITH AFTER-PROTRUSION OF THE INTESTINES, AND FORMATION OF AN ARTIFICIAL ANUS.

The following history of a very curious and exceedingly rare pathological process was communicated to the much respected editor of the Journal, which in this number we have often had occasion to name, (Rust's Magazin für die gesammte Heilkunde) by Dr. WEESE of Thorn, who collected the particulars of the case from the most authentic sources.

Gorecka, a Polish peasant's wife, residing at Orzectrowo, a village about three miles distant from Thorn, now about 47 years of age, was, in the year 1807, pregnant with her tenth child. She passed through the pregnancy without any thing particular occurring, the labour commenced at the expected time; the pains were regular and strong, and the membranes broke early. The labour, however, did not go on, and after violent and repeated efforts, the patient felt as if something had suddenly burst internally, and, at the same time, a small quantity of blood was discharged from the vagina. The labour pains ceased, and in this state a physician was sent for from Thorn, who having made the necessary examination, gave it as his opinion, that the child was adhering to some part of the abdomen, and that the woman could not be delivered. This person appears to have been a quack, as his name was unknown to the regular practitioner of the neighbourhood. In this state of obscurity the case was left, and the poor woman resigned herself to her fate. The motions of the child, which had been before very distinct, had, according to the account of the patient, ceased at the time of the accident before mentioned; she was obliged to lie in bed; the tumour of the abdomen, which was very hard, began in a few days to be painful, and produced a circumscribed swelling in the belly, which gradually burst in the umbilical region. The minutiæ of the case are not easy to collect, but it appears that no farther medical aid was sought for; that the suppuration went on, and that the husband became the surgeon and daily dressed the wound, cleaned it from the copious discharge of matter, and eventually drew out several pieces of bone as they appeared in the wound. The memory of the patient could not furnish the information whether any portion of intestine was pulled out in the attempts made to extricate the pieces of bone, or whether a portion of the intestines protruded through an opening left in the part after all the remains of the fœtus had been discharged, or if the intestine had come through by any unusual effort after the wound had entirely cicatrized. She said, however, that a piece of the intestine came through and afterwards increased in size, that it opened and discharged fæces; that the discharge per anum began to diminish as the unnatural outlet enlarged itself, and after a time entirely ceased.

We come now to a more satisfactory part of the case as described by Dr. Weese from actual observation. In the middle point of the abdomen, in which every trace of the umbilicus is lost as well as the usual rotundity of the part, is a circumscribed, irregular, and somewhat circular projection, with sharply defined edges, from between which wound two separated portions of prolapsed intestine protrude; the greater turns downwards from left to right, describing almost the figure of an Italic *s*. The plate which accompanies the description of the present state of the patient shows that the prolapsed intestines are of a bright red colour, and the edges of each portion of intestine appear to have coalesced with the surrounding skin, so that there is no distinct separation between them. The peristaltic motion of the intestine was, on several occasions, distinctly observed by Dr. Weese, and, he remarks, that the outer coat of the intestine was much altered in texture, so that it appeared like the newly-formed skin of a cicatrix. The larger piece of intestine measured fourteen inches and six lines (Prussian measure) in length, and the other ten; each piece was in several places from two to three inches in breadth. They both appear to come out of the abdomen at a common opening, and each of them is furnished with an opening large enough to admit of the finger being introduced into their cavities. The exit of the fæcal matter is easy, and generally comes out in small quantities, which are received on pieces of cloth. The only support or protection afforded to the part is made by folding a piece of *coarse sackcloth* over it, and applying a moderate compress of the same material above. The patient has lived fifteen years with this frightful protrusion, and has, during the greatest part of the time, enjoyed good health. She not only is able to attend to the affairs of her house, but also to work in the garden, and was seen by Dr. Weese busily engaged digging the ground for potatoes. She had, up to the preceding year, regularly menstruated, and was, even to the time of the drawing up of this case, in good health.

---

### 15. THE HUNGER-CURE; AN ACCOUNT OF TWO CASES IN WHICH IT WAS SUCCESSFULLY EMPLOYED.

Dr. Suttinger, in Kosten, has published two cases in which he used the hunger-cure with success. The first case was that of a girl about thirteen, tall, but not well shaped. In her seventh year, she had, for the first time, scrofulous ulcers and caries, which, from having appeared in various parts of the body, very much disfigured her person. The one arm was shorter than the other and bent; the left eye-lid was shortened by a large cicatrix, so that she could not cover the surface of the eye. For about two years the girl was pretty well, but at the end of that time, a sore broke out on the edge of the hard palate which soon affected the bone, and extended far into the nostril, by which the inferior spongy bone soon became carious also. All the usually recommended anti-scrophulous medicines were tried without any avail, bark, iron, mineral acids, baths, and so on. Mercury was also given to salivation, but did not stop the progress of the disease. Several holes had formed in the palate, and the stench of the discharge was horribly offensive. On account of the inertness of the former plans of procedure, the hunger-cure, without mercurial friction, was proposed, modified a little to meet the circumstances of the patient. The process was managed in the following manner. Three times in the day, at nine, twelve, and five, was a small plate of milk gruel allowed, but not sufficient to satisfy the appetite; for the ordinary drink, the decoction of the woods was given, and mornings and evenings, four six-grain pills of the *extractum cicutæ*, with the powder, were prescribed. All other sustenance was most strictly forbidden. The success surpassed expectation. After a few weeks, the ulcers had lost their unpleasant smell; their edges began to look clean; the matter ceased to be discharged from the nose, and, after eight months more, all the sores completely healed and cicatrized. A small opening only remained in the palate. The patient remained for several months after quite well, but has not as yet menstruated.

*Case* 2. A second case, in which the same plan of treatment was observed, was quite successful. This patient was also a girl, but of a full plethoric habit. She had for many years a severe and obstinate eruption on the face, but particularly on the nose and upper lip, where an ulcer formed, which began to assume a malignant character. The usual treatment employed in such cases was tried without any benefit. The same allowance was given to this girl as to the former, and the same medicine, which, after five weeks, ended in acomplete cure. —*Rust's Magazin für die gesammte Heilkunde.*

16. CASE IN WHICH A NUMBER OF LIVING WORMS WERE VOIDED FROM A CHILD'S BLADDER.

A child, two years of age, who had been for a long time sickly, became very much emaciated and subject to sudden spasms in the bowels, without the practitioner's being able to discover the cause of the ailment. Some Vienna drink was given to the child, under a supposition that the mesenteric glands were enlarged. A soap liniment was directed, at the same time, to be rubbed on the back and belly, and a solution of the acetas potassæ was given in marshmallow tea. Two days after this treatment was adopted, the father perceived in the child's urine four living worms, which, for some little time, appeared very active and then died. The next day more were discharged, they were then examined by the physician who attended the child, and he describes them to be very much like the larvæ of the common meat fly. There were two dark-coloured spots on the head; the tip of the tail of each larva was also dark. Their usual length was from four to five lines long, and breadth one. These worms continued to be discharged for twenty-one days, in a greater or less number daily, and it was found that their expulsion was assisted by giving the child pretty strong doses of camphor mixture.—*Rust's Magazin.*

17. PROSOPALGIE* CURED BY CAUSTIC.†

The following case obstinately resisted the combined influences of the whole class of narcotics, blisters, and metallic oxyds, with which the patient was vigorously treated for eight months. If further experiments should confirm the utility of the caustic, it will prove a valuable addition to our therapœia.

Madame N. had, during three years, suffered at certain intervals a violent pain in the face. At first the pain returned only once in the month, but, in the last year, it renewed its attacks, and these very violently, every week. The pain, in each of the paroxysms, pursued a regular course without any warning or precursory symptoms; it commenced in the lower jaw, as if many burning needles were stuck into the neighbourhood of the part in which the *nervus maxillarius inferior*

† *Journal der practischen Heilkunde: November.*

emerges from the *foramen mentale.* This violent pricking pain darted like lightning over the right half of the lower jaw, upper jaw, cheek, and extended to the upper and lower eye-lid of the right side. The left half of the face remained quite free from pain. With the extension of the pain over these parts, its intensity momentarily increased to a very high degree; the least motion of the parts made the pain, if possible, still worse. After this paroxysm had continued four, six, eight, and even sometimes twelve hours, it began gradually to subside from the periphery to the point from which it commenced, so that the last trace of pain was felt in the right mental foramen.

Between the angle of the lower jaw and mastoid process of the temporal bone of the right side, a plaster was put on, in which a small opening of about the size of a half silver groschen,* was made, and a paste of the *lapis causticus* introduced. This was secured in the usual manner and allowed to remain three quarters of an hour. When the eschar was thrown off, a pea was put into the hollow to keep up the suppuration. The reason of the practitioner applying his caustic to this part was, that he might immediately act upon those branches of the pes anserinus which accompany the nervus alveolaris inferior to the mental foramen. Three days after the application of the caustic, a violent paroxysm of pain returned, during which the patient was so unmanageable that she tore off all the applications to the part, so that it became necessary to encourage the suppuration by the use of the mezerion powder. During the two following weeks, two mild paroxysms returned, which continued only a half minute. Since that time to the present, (about nine months) the complaint is so diminished that the patient feels only now and then, when the weather is damp or rainy, a trifling uneasy sensation in the cheek, which remains only for a moment.

At the conclusion of this case Hufeland makes the following remark —I cannot allow this opportunity to go by without observing, that I have many times found that counter-irritants and issues, applied in the space which lies between the flap of the ear and the mastoid process, have been the most effective in chronic cases of ophthalmia and for pains in the ear, teeth, and head; and I am quite satisfied that a small issue or irritated surface does more good in this situation than large ones elsewhere, as, for example, in the neck and temples.

---

18. CONCEPTIO EXTRA-UTERINA.†

Dr. Stephany, of Frankenstein, has, in the Reports cited below, described the following case of extra-uterine pregnancy.

The wife of the peasant Franz Rimplen, at Landel, 43 years of age, the mother of three sons, with each of whom she had easy labours, be-

---

* Half as large as our sixpence.

† *Miscellen Preussischer aerzte aus den vierteljährigen Sanit ätsberichten.*

came pregnant about Michaelmas, 1815, for the last time. She expected to be confined about midsummer, and during her pregnancy continued to enjoy good health. Three weeks, however, before the time of her expected confinement, she leaped over a ditch, and heard, in the act of doing so, a loud crack in her abdomen; this was almost immediately followed by discharge of blood and general uneasiness. During the hæmorrhage, which continued about 14 days, the belly gradually lost its regular rotundity, and there formed gradually in the right side a round hard tumour which occasioned pain on every motion. Notwithstanding this, she is said to have enjoyed pretty good health until the September of 1822, when she was attacked by fever, followed by great depression of strength; and, in October of the same year, some portions of bone were discharged by the anus. The patient gradually became worse and in the month of July, 1823, she died. Dr. Stephany was called to her a few weeks before her death, after many other physicians and surgeons had been consulted. He found the patient in a state of great debility, with considerable irritation, fever and pain; he instituted an examination, per anum, and discovered in the upper part of the intestinum rectum an opening through which, or in which, a piece of bone could be felt. The pieces of bone which had been in this manner, from time to time voided, were collected by Dr. Wenzel, and shown to Dr. Stephany; they were the bones of a fœtus almost full grown, and very discoloured.

*Inspectio Cadaveris.* The examination of the body, which was made in the presence of several physicians, gave the following results:—The abdomen externally, appeared quite natural; the whole body was much emaciated. The viscera were found in their natural situation; but the intestines were much inflated. The upper surface of the liver was united to the cartilages of the ribs, and the under surface was adherent to the stomach. The colon on the right side was much distended, and, on the left, thickened, and for about two inches in length, very much drawn together. The *vasa meseraica* were filled with blood, the mesentery itself unnaturally thickened and adherent in many places to the surface of the intestines. The stomach small and contracted, the descending colon a little distended and united to the spleen, and, from that place to the rectum it was fast drawn together. The cæcum was firmly united to the rectum. In one of the deep convolutions of the colon was a sac-like, distinctly defined swelling, with all the surrounding parts firmly connected, and about fifteen inches from the end of the ileum. Its outer surface was of the natural colour, the inner, speckled. It contained the remaining portions of the three broad bones of the cranium, these were, for the most part, of a horny texture, of a black colour, and the edges were very much thinned and corroded. This sacciform swelling extended itself posteriorly to the rectum with which it communicated by the opening before named, through which the finger could be readily passed into that intestine, both upwards and downwards. The *tuba* and the *ovarium* on the left side were quite natural; the tube on the right side much enlarged, hardened, and degenerated; the orificium *uteri internum* closed; the right ovary was also destroyed, and, on the inner side of the fallopian tube, were here and there several pieces of bone. The *uterus was in every respect healthy:* but the os sacrum was, on its internal surface, in a carious state.

---

### 19. CUTANEOUS MEDICATION.*

We often choose to gather our honey among thorns that prick us pretty sharply. The pleasures of the table are not unfrequently alloyed by the bitter task of swallowing drugs afterwards—and the *stomach* is the organ which pays the penalty of its own pampering. This is a specimen of that retributive justice which Nature seldom fails to administer, when her laws are violated. Man, however, is always exciting his ingenuity to evade the penalties of the law, whoever may be the legislator. Dr. Lesieur has proposed to transfer the disagreeable duty of taking physic, from the stomach to the *skin*—the latter organ being far less fastidious in its tastes and antipathies than the former. This will be glorious news for the gourmands, and must make Dr. Kitchener's heart rejoice! We apprehend, however, that the "Medication Endermique" will not prove quite so palatable to the practitioner as to the patient. The mouths of the cutaneous absorbents, though numerous, are not of calibre to admit the "*quantum suff.*" of pills, powders, and potions, according to the present system of medical remuneration. But as that system is about to undergo a parliamentary investigation, we hope the Honourable Member for Aberdeen will be prepared with an accurate calculation of the immense saving which must result to the nation, in phials, corks, and pill-boxes, by the new or *endermic* practice of physic.

Seriously, we do not anticipate, with M. Lesieur and the commission who are ordered to test his experiments, that cutaneous medication can ever be carried to any great extent. The absorbents on the surface are very uncertain agents. We can, it is true, impregnate the system by mercurial inunction, or lull the sensibilities of the nerves by opiate frictions;—but we may rub in whole ounces of emetic tartar on the skin without producing the least disposition to sickness of the stomach. The author of the endermic system was aware of this, and therefore is obliged to apply his remedies through the medium of a *blistered surface*—a condition which, on many accounts, will for ever prevent the extension of cutaneous medication. In most of the acute diseases, we dare not wait for denudation of the cutis—nor trust to such a fickle process as absorption for the inhibition of active remedies. In chronic diseases, on the other hand, it can but rarely happen that such a *quantity* of medicine is necessary, as to render it any great object to save the

---

* Nouvelle Medication par la Voie de la Peau, &c. Par M. Lesieur, M.D.—*Archives, Juin,* 1826.

stomach from the disagreeable reception of drugs. Still there may be cases, where gastric irritability or other circumstances make it desirable to introduce medicinal agents through the external surface of the body, and, therefore, practitioners should be aware of the amount of power or facility they possess for this purpose.

In the 9th number of this series, p. 252, we gave an instance of the beneficial influence of acetate of morphine, applied to the denuded surface of a blister, by M. Serres at La Pitié; and it appears that the majority of M. Lesieur's experiments were on the same medicinal substance. Seventeen cases, in all, are related by our author, as treated at the Hospital Cochin, and the Bicêtre. Of these, four were chronic catarrhal affections, which were promptly relieved, and soon cured by the external application of acetate of morphine to a blistered surface. The quantity was, at first, half a grain, gradually increased to two grains. The medication was continued a month.—At any time, when the application was intermitted, the symptoms returned. Two cases of phthisis pulmonalis were much relieved by a similar application. In this disease, and in all diseases of structure in the lungs, the dose must be smaller than that which is above stated. The next case was one of pleurodyne, which had resisted leeches, blisters, and other remedies, but gave way to the morphine externally applied. A neuralgic affection of the temple was cured also by the same means. In the ninth case, powdered strychnine (one sixth of a grain) was applied on a blistered surface, for hemiplegia. The quantity being increased to two grains, a *paroxysm of tetanus* supervened, but was dissipated by the subduction of the strychnine and the substitution of acetate of morphine. This last circumstance induces M. Lesieur to propose the application of morphine in idiopathic or traumatic tetanus itself. The remaining cases relate to the endermic method of employing sulphate of quinine, musk, and emetic tartar. Our author has cured intermittent fevers of different types—convulsive cough—has raised perspirations—re-established and checked expectoration, &c. &c. by the proper remedies applied to the denuded surfaces of blisters. The Royal Academy of Medicine have been so taken with the apparent utility and importance of the "medication endermique," that they have appointed Messrs. Andral, Double, Chomel, and Segalas, to test this process of curing diseases, by clinical experiments. We shall hear, in due time, the result of this examination.—In the interim, some of our medical officers of public institutions might easily try the effects of this method, and report upon it in the periodical journals.

---

### 20. AMPUTATION OF THE LOWER JAW.*

In the first volume of the present series, page 213, we gave an account of an amputation of the lower jaw, performed by Professor Lallemand, of Montpellier—and in the second volume of the same series, p. 210,

* Dr. M'Clellan. American Medical Review, vol. ii, p. 152, *et seq.*

we gave the details of a similar operation by Dr. M'Clelland. In a late number of the American Medical Review, Dr. M'Clelland has drawn the attention of his brethren to "the reproduction of bones," and to a "recent case of amputation of the lower jaw," with observations on the propriety of repeating similar operations without previously securing the carotid arteries.

Since the publication of his first case, Dr. M'C. has met with several others, which he deems of sufficient interest to lay before the profession. Of these we shall give a short account in this article.

*Case* 1. A little girl, three years of age, had been afflicted for several months with a horrible ulceration of the gums and cheek, which had been arrested by Dr. Eberle, the whole inferior maxilla, however, being left in a state of necrosis, and the surrounding soft parts so completely detached from it, that its size alone prevented it from being extracted from the mouth. By pushing the lower jaw downwards they were able to expose the whole front of the jaw, which they divided with a saw at the symphysis, and extracted the two halves entirely, with the exception of one of the condyles. The cavity was stuffed with lint, and the chin lightly supported by a few turns of the head-bandage. In the course of a few days, granulations sprouted up most luxuriously—filled the cavity previously occupied by bone—and were completely cicatrized over by an extension of the mucous membrane. About two years afterwards Dr. M'C. accidentally fell in with his former little patient, and was surprised to find the countenance so natural, and the motion of the lips and chin so perfect, that no one, unacquainted with the history of the case, would have supposed that any part of the maxilla had ever been removed. To the sense of touch, also, the parts presented a firm and well-defined bony resistance; and Dr. M'C. was informed that the child could masticate her food and articulate her words with ease and perfection. On examination, however, it was found (as might naturally be expected) that the lower jaw was entirely destitute of teeth, their place being occupied by a cicatrix of the firmness of cartilage; "and, on inspecting the conformation of the new bone a little more carefully, the angles were found to be deficient, or rounded off like those of a new-born child." This case, our author thinks, affords decisive evidence "in favour of the probability of a reproduction of osseous matter, after the removal of almost any bone." Dr. M'C. supports this assumption by some other instances that have occurred in the practice of surgeons. Dr. Whitridge relates the case of a young soldier, in which nearly one half of the inferior maxilla was extracted in a necrosed state, a restoration of the lost substance afterwards taking place, and the patient being left with a useful jaw. Decker, in his Exercitationes Practicæ, describes a case in which full two-thirds of the same bone were taken away, and where reproduction appears to have been effected by Nature. In the Memoirs of the Royal Academy of Surgery, there are other cases of a similar kind described;—and a case lately occurred in the practice of Professor Mussey, of New Hampshire, in which the scapula was partially regenerated, after having been torn out from the body by a piece of machinery into which the patient had fallen. This case is very similar to the celebrated one of Chopart, in which the scapula was partially reproduced after having been entirely destroyed by necrosis.

In respect to the precaution of tying the carotid artery, before attempting any formidable operation about the throat and jaw, Dr. M'Clelland decidedly disapproves of this measure, as greatly increasing the sufferings of the patient and the danger of the operation. Dr. M'C. has extirpated almost all the glands about the throat in succession—the lower portion of the parotid, and many of the lymphatic glands—he has repeatedly exposed the carotid artery and jugular vein—and he has even dissected away tumours from the very coats of these vessels, without encountering immediate danger or subsequent inconvenience. Finally, he has recently performed an operation, which is supposed to require previous ligature of the carotid artery, and of which we shall now lay the particulars before our readers.

*Case.* Mr. Brown had an enormous carcinomatous tumour, occupying the lower part of his right cheek, and extending over a large portion of the throat. It commenced on the under lip, in the year 1820, and gradually extended downwards and backwards over the base of the jaw. It was circumscribed—and presented a firm cartilaginous structure. The upper portion of this tumour had been ulcerated for several months, and was now discharging an offensive ichor. On examination by a probe, the subjacent bone was found to be carious. Severe lancinating pains had long been experienced by the patient—his appetite was impaired—but his health was otherwise good. He was only 42 years of age, and anxiously desired an operation.—The steps of this bold surgical process, we shall give in the operator's own words.

"On the 28th of July, I performed an operation for his relief, in the hall of the infirmary of Jefferson Medical College, in presence of the professors and pupils of that institution. An incision was commenced at the left commissure of the lips, and carried downwards, over the symphysis of the jaw and right side of the thyroid cartilage, to the anterior edge of the sterno-mastoid muscle. A second incision was next carried from a little above the right commissure of the lips, around the opposite margin of the tumour, and across the angle of the jaw, until it reached the termination of the former incision. The whole mass of the tumour was then dissected downwards from the surface of the jaw, and deeply from the throat beneath, until all the parts that were included between the two incisions had been removed. By this dissection several enlarged lymphatic glands were removed from the throat, together with the original tumour on the cheek.

"After securing the facial and coronary arteries, I next proceeded to examine the condition of the jaw, which was deeply affected with caries. Having satisfied my assistants that it was absolutely necessary to remove it, I divided it by the metacarpal saw in two places,—1st, at the symphysis, between the two front incisors, and 2dly, a little before the angle—between the second and third molares. On dissecting out the intermediate piece of bone, the facial artery was again divided and secured; after which three more indurated, although not much enlarged, lymphatic glands were removed from the throat. As the surface of the sub-maxillary gland appeared to be discoloured and tumefied, all that portion of it which lay on the lower surface of the mylo-hyoideus muscle was removed;—the remainder, which accompanied the excretory duct above the same muscle, being evidently sound, was left undisturbed. Of course the facial artery was again laid open by this part of the dissection and secured, for the third time, much nearer its origin than before. Although the sublingual gland was so fully exposed that it protruded very much, it was not deemed necessary to excise it, on account of its healthy appearance.

"The soft parts being now satisfactorily disposed of, I proceeded to examine the posterior extremity of the bone, the cancellated structure of which appeared somewhat discoloured, and bled profusely. As its condition was so suspicious, and as it moreover formed an inconvenient projection, which the integuments could not be made to cover, I applied the saw again, just at the angle, and removed about an inch more of the bone, together with the last molar tooth which was implanted in it. The integuments were then approximated, as much as possible, by three interrupted sutures, by means of which the cut extremities of bone were brought much nearer together than before, and the size of the wound, also, was greatly diminished. Some strips of adhesive plaster, and pledgits of patent lint, secured by a head bandage, completed the dressing, after which the patient was able to walk about with great ease." 163.

As the sutures produced painful tension, and displaced the two portions of bone, Dr. M'C. removed them on the second day, and trusted to adhesive plaster. From that time no particular pain was complained of, and the patient continued to recover rapidly. In less than ten days the cavity in the throat was entirely closed, and in three weeks the patient was so nearly recovered, that he insisted on returning to his family. The account only reaches to the 2d September, at which time the wound was nearly healed, and he had the full power of mastication and articulation.

Dr. M'Clellan expresses his surprise that surgeons should ever have thought of anticipating hæmorrhage by securing the common carotids, in operations about the face and neck. The anastomoses between the branches of all the main arteries of the head are so large and frequent, that he questions whether the division of any one of them would not be attended with a troublesome hæmorrhage, even after the closure of both carotids. All vessels of any size that are cut, must, and may, be easily tied, at the time of division, and this is all that is necessary.

---

21. NEURALGIA.

So many speculations have been hazarded respecting the *nature* of this dreadful disease, and so little has been ascertained of its pathology, that we are not very eager to listen to a new theory on the occasion. Dr. Trevor, of New York, has published what we believe to be a new view of tic douloureux, connected with a novel mode of treatment, which we shall briefly notice. He avers, that neuralgia has seldom been known to occur, except in parts of a dense fascial or aponeurotic texture, and he views the disease "as an inflamed state of the periosteum of the bones, to which the nerves involved in this disease are distributed." It is this inflammation, he thinks, which causes the irritation of the nerves. The management of the complaint he arranges under three heads—*first*, where it occurs in people of sound health—*secondly*, in plethoric habits, with determination of blood to the part—*thirdly*, in people of nervous temperament, whether this be the effect of the disease, or of original constitution. The treatment of the first class is very simple—the obvious and most speedy remedy being to lay the inflamed part freely open with a lancet. "Where the cheek, the most general seat of the disorder, is affected, the operation is performed from under the lip, thus doing away the necessity of causing the unsightly appearance of scars on the face. The part to be operated on, is that from which the nervous twitches commence, and to which the patients will almost always direct your attention." If these indications do not exist, as is sometimes the case, then the proper place may be found by passing the finger under the lip, and pressing with some degree of force on the periosteum of the maxillary or molar bones. The moment the diseased part is touched, the patient exclaims, and sometimes by this alone the convulsive twitches are excited. At this spot Dr. Trevor incises the periosteum, freely and extensively. The operation is attended with considerable pain; but in the course of half an hour this inconvenience vanishes. If the disease returns, the operation must be repeated.

In the second class of patients, the system must be reduced to a natural and healthy standard, otherwise the local operation will give but temporary relief. The means of doing this are obvious.

In regard to those of the third class, (the most numerous of all) whose nervous system is in a state of derangement, as well as that of the digestive organs, our author advises the operation as the first step, and then to take off the morbid sensibility of the nerves, by tonics, and especially by the carbonate of iron, paying due attention to the chylopoietic apparatus.

Several cases are detailed by Dr. T. in illustration of these modes of treatment; and we need hardly say that, in so dreadful a disease as this, we must be thankful for any hint that promises to allay the sufferings of our patients, and rescue medicine from the charge of inefficacy.—*North American Medical and Surgical Journal, No. 2.*

---

22. LITHONTRIPTIC PROCESS.*

It is evident that, although M. Civiale's "Lithontripteur" has had little or no success in this country, it has been otherwise in the hands of the inventor. An army physician, Dr. Brousseaud, has been, among others, successfully operated on by M. Civiale, and has laid his case before the Academy of Medicine, in justice as well as in gratitude to his deliverer. We shall briefly state the particulars.

---

* Dr. Brousseaud. Archives Generales, Avril, 1826.

In the month of September, 1824, Dr. B. began to experience symptoms of calculus in the bladder, and in February, 1825, he passed one, while making water, which was three lines in diameter, and weighed nine grains. It presented unequivocal evidence that there were others left behind. He continued to suffer severely till the month of June, when he applied to M. Civiale, who sounded him, and detected the presence of several calculi in the bladder. The process of dilating the urethra was then commenced, and on the 2d of July, the "LITHONTRIPTEUR" was introduced, for the first time, into the bladder, and a calculus, (which was judged to be about seven lines* in diameter) seized, and in eight minutes broken into fragments, of which Dr. B. passed, in the course of the next 48 hours, 36 grains in weight. For some days after this operation, the urine, which was passed more copiously and with much pain, was strongly tinged with blood. Quietude, low living, and hip-baths dissipated these symptoms; and, on the 7th of July, the instrument was again introduced. Ten minutes were spent in seizing and crushing several fragments of calculus, of which 26 grains, in a state of detritus, were passed in the course of that and the succeeding day.—The third introduction was on the 12th July, and was attended with much pain and efforts to make water, caused, most probably, by the searching for small fragments of calculus. Twenty-three grains of detritus sabulosus were voided after this operation, and considerable pain was experienced in the loins, the urine being charged with blood for some days subsequently. A fourth introduction took place on the 19th July, and was followed by the discharge of 24 grains of detritus, and much less inconvenience in making water than on former occasions. 27*th July*. The operation this time was attended with very little pain, and only 14 grains of detritus came away. On the 3d of August, the sixth and last exploration was made by M. Civiale, and ten grains of detritus were discharged on the same day, without any inconvenience. From this time Dr. B, recovered progressively, his urine becoming clear and easily passed, and his general health improving. He could now travel in a carriage, without experiencing any uneasiness about the bladder. In the course of these six operations, 130 grains of calculus (principally uric acid) had been broken down and discharged. Messrs. Lacroix, Debaltz, Baibette, Wesley, Humphreys, and Robinet, witnessed these operations, and the results above stated.

Before Dr. Brousseaud submitted to the operation, he was desirous of seeing it performed on some others; and, in this, he was gratified by M. Civiale. Two patients underwent the lithontriptic process in his presence. The first was a youth of 19 years, who had the instrument introduced eight times, at short intervals, by which a voluminous calculus of oxalate of lime was broken down and evacuated. The second patient was a Sexagenarian, who had been, for several years, tormented by calculi in the bladder. Four introductions were sufficient to rid him of his evils, the calculi being very friable.

* The French line is one twelfth of an inch.—*Rev.*

In the report which M. Roux, M. Cloquet, and M. Hervey have made to the Academy, they remark that it is incontestibly proved that a calculus of small volume may be broken in the bladder, and the fragments passed off, by the urethra, without any division of parts, and by means of a *straight* instrument. And, they observe, although this operation must be repeated a certain number of times, and is not unattended with pain, yet it is far from involving the same chances of danger that inevitably attach to lithotomy. One of the reporters was present at five operations of M. Civiale, and expresses his highest approbation of the operation and of the dexterity of the operator.

---

23. CASE OF CROUP RELIEVED BY THE EXPULSION FROM THE LARYNX OF A FALSE MEMBRANE OF SOME EXTENT.*

The subject of this paper was a child, seven years of age, under the care of Dr. Monronval the narrator of the case.

On the 1st October, 1824, the little patient felt chills in the loins with great general *malaise*. By the next day the submaxillary glands were swollen, the larynx painful, and there was some difficulty in swallowing the saliva, &c. October 3d. All these symptoms were exasperated; there were, besides, a frequent and painful cough, obstructed respiration, the tonsils swollen and covered with a whitish film, deep reddening of the velum palati and posterior part of the pharynx, quick small pulse, fever, and constipation. Venesection, 10 leeches to the larynx, sinapisms, lavements, &c. &c. were ordered, but at night there was severe exacerbation. 4th. The difficulty of swallowing less, cough incessant and croupy, eight leeches, &c. 5th. Worse than before. 6th. The saliva contained several portions of false membrane: cough violent and convulsive, delirium at times. Twelve leeches were applied to the neck and lavements, &c. given in abundance. 8th. Croup still extremely severe. In a very violent paroxysm the patient coughed out a membranous tube about 11 lines in length and one line in breadth; its form was an irregular ring, cleft on one side, and when pressed between the fingers it was readily broken down. It was an exact cast of the commencement of the larynx. Immediately from the expulsion of this membrane the child began to recover. The cough abated, respiration was freer, the tonsils resumed their natural size, appetite returned, so did sleep, in short, on the 14th, convalescence was established; the boy has, however, been always since very subject to attacks of sore throat and cold on the slightest exposure.

---

24. EPILEPSY FROM TÆNIA.†

Irritation of the gastric and intestinal nerves produces a host of physical and intellectual phenomena that are little suspected by the routine

---

* Journal Complémentaire, April, 1826.

† Sur quelques Maladies graves Guéries par l'Expulsion du Ténia. Par M. Gaube.—*Rev. Med. Juillet*, 1826.

practitioner; and we are convinced that the study of this irritation opens the widest and the most fertile field for the talented physician, of any that lies in his path at the present moment. We are not without hope that something interesting will, ere long, appear on this subject.

*Case* 1. Bonet, aged 34 years, of strong constitution, had enjoyed good health till the age of 19; at which epoch he experienced a first attack of epilepsy, without any apparent cause. The subsequent accessions became more and more frequent, till at length he had them weekly, and yet without any preceding affection of the head. Several physicians were consulted, and various remedies were tried, and without any benefit. Afterwards it was observed that the patient passed by stool some joints of tænia. The means they employed to expel the worm were also unsuccessful. In 1818, when he consulted M. Gaube, he had, besides the usual severe attack, some accessions daily, of a milder kind, and which, though they did not cause him to fall to the ground, deprived him for half a minute, or so, of his faculties. At this time the following curious phenomena were observed as precursors to the regular attacks, viz. A sudden difficulty of speaking, quickly succeeded by a sense of spasmodic constriction in the right side of the neck, ascending from its basis along the œsophagus, apparently, to the tip of the tongue, which organ was drawn to the right side at the moment. Sometimes the patient had sufficient warning to endeavour to catch hold of any furniture or other object within his reach, before the fall, which was always on the right side of his body. The paroxysm lasted from ten to fifteen minutes, during which, the anterior part of his neck continued very much swollen. After each attack he felt, for an hour or two, a slight pain over the orbit of one of his eyes. He was generally able to go to work immediately after the paroxysms.

Our author, suspecting the irritation of tænia to be the cause of the epilepsy, tried various remedies recommended for the expulsion of that worm, and with the effect of dislodging a very large quantity, but without evidence of the head or any part of the neck being included in the portions expelled. Bonet's health improved after this, and he experienced an immunity from any severe paroxysms of epilepsy for the space of six months, when the attacks returned as violent and as frequent as ever.

In 1823, Bonet was seized with a very severe inflammatory disease, commencing as a violent sciatica, and afterwards taking the form of enteritis, which reduced him to a very low ebb, and ended with symptoms of ascites. This illness lasted four months, during which he was free from epileptic seizures. In the course of a few months, after his recovery, the epilepsy returned, and was attended with occasional discharges of some portions of the worm. On the second of June, 1825, M. Gaube administered the decoction of pomegranate bark (two ounces boiled in a pint and a half of water to a pint, and taken at four draughts, in the course of the morning, fasting) which brought away several fragments of the worm. On the 3d, The same dose, and the same result. On the 4th, The medicine was repeated, when 22 feet of worm were ejected in one unbroken piece, including the head of the animal. From this time Bonet experienced no more epileptic paroxysms, and his health is perfectly re-established.

*Remarks.* The efficacy of the pomegranate bark in expelling tape-worm is now, we think, established beyond all doubt, by physicians in various parts of Europe; and as it may be procured in abundance from Mr. Butler, of Covent-garden, we hope soon to have the testimony of English practitioners in its favour.

The suspension of the epileptic seizures during the existence of the acute diseases, in the above case, and for some time after convalescence, is not unworthy of notice, and may furnish some useful matter of reflexion to the pathologist. The duration of epilepsy for fifteen years without any material deterioration of the intellectual function is remarkable, and can only be accounted for by the cause of the epilepsy being at a distance from the sensorium, which was only sympathetically affected. Indeed, the curability of this distressing disease generally depends on its being symptomatic of irritation in some of the viscera, and we are strongly inclined to think that the lunar caustic is principally successful where the paroxysms are dependent on *intestinal irritation*, which this medicine is peculiarly efficacious in removing, by diminishing *irritability* of the visceral nerves. We had lately a remarkable instance under our care, where a course of three months of this medicine cured a most distressing spasmodic affection of various muscles, that had resisted every remedy for a number of years. We suspected the cause to be morbid sensibility of the gastric and intestinal nerves, and the event fully justified our suspicions, and more than realized our hopes. We shall give the case more at large in a future number of this Journal.

*Case 2.* Ferguson, 39 years of age, an English seaman, and then a prisoner of war, entered the Military Hospital of Metz, on the 30th September, 1811, for a severe hæmaturia, preceded and accompanied by abdominal pains and progressive emaciation. The hæmaturia had been of three weeks duration, the abdominal pains of nine months' standing. Eating frequently gave the only relief to the pains. The patient said that, some weeks previously, he had passed something like a flat worm, soon after which the hæmaturia commenced. The whole of the abdomen was painful on pressure. Suspecting the presence of tænia, M. Gaube prescribed anthelmintic medicines, which were followed by a considerable diminution of the pains and of the bloody discharge. On the seventh day of treatment a strong anthelmintic, followed by a drastic purgative, brought away four joints of a tape-worm, each nearly an inch in length. The male fern was then administered, and the hæmaturia and abdominal pains were still farther mitigated. Several doses more of the medicine were given, with suitable cathartics, and immense quantities of the worm were, in consequence, discharged. The whole of the symptoms disappeared, and the sailor was discharged the hospital cured on the 25th November.

This is a curious instance of the effects of intestinal irritation, and well worthy of record.

---

25. SPECIFIC DISEASES AND MEDICINES.*

In his preliminary observations, Mr. Allan makes several pertinent remarks on the importance of "carefully discriminating between those *minute shades of difference* in the features of diseases, and the operation of remedies," which, to a careless observer, appear unimportant. The utility of this discrimination we grant—but we are sorry to observe that, in our journey through life, we have sometimes found the phantasies of an ardent imagination depicted, and even embodied into forms of shadowy existence, which served to puzzle and bewilder, rather than enlighten our path at the bed-side of sickness. We are advocates for accurate observation of the phenomena of diseases:—But we may perchance refine too far. Our attention may be so rivetted on minute and evanescent shades of distinction, of little real importance, that we may over-look those broad characteristics, which are patent to the eye of common observation, and which prove the only safe land-marks to guide us in the moment of difficulty and danger. A microscope would be a sorry instrument through which a general might reconnoitre the positions, or watch the manœuvres of his enemy in the day of battle. Mr. Allan we know to be a judicious practitioner, as well as an accurate observer; but we cannot help considering the following qualification in the physician, as Utopian. "The accomplished physician, says he, ought to be capable of weighing with accuracy all the circumstances of the case, and of selecting out of whatever class of medicines may be indicated, *the individual article precisely adapted to the peculiarities of his patient's case.*" How is this to be done, without first knowing the *peculiarities* of his patient's constitution? The *precise* article for the complaint may not be the *precise* article for the patient. What will vomit one person, will purge another—act on the skin of a third—and produce no sensible effect on a fourth. This *precision of adaptation*, therefore, is too much a matter of chance to be always expected from a physician of the most consummate skill, or unlimited experience. Let us grasp what is within the reach of human power, and we shall succeed better than by aiming at perfection, which is not the lot of humanity. But to business.

The present paper of Mr. Allan's is presented to the profession with the view of illustrating the property which cinchona has long been known to possess as an antidote to periodical diseases. In this there can be no novelty, of course;—but the mode of administration claims our notice. Five cases are detailed by Mr. Allan, as examples of the efficacy of small doses of bark. We shall sketch three or four of these, for the benefit of our readers.

*Case* 1. A man of sober habits was attacked with a painful affection of one ankle, without any swelling or discoloration, which came

---

* Mr. Allan, Med. and Phys. Journal, Aug. 1826.

on every night, after going to bed, and arrived at an agonizing degree of severity by three o'clock in the morning. Mr. Allan commenced by smart purgation with the compound colocynth pill, and then administered *ten grains* of powdered bark every three hours, the bowels being kept open by smaller doses of the above-mentioned purgative. The pain ceased after he had taken a dozen of powders;—But, another dozen was recommended as a security against relapse. The complaint did not return.

*Case* 2. A servant had been exposed to wet and cold on his passage from Aberdeen to London in a smack, and soon afterwards became affected with pains in his shin bones—first in the one, and ultimately in both. The pains observed regular diurnal periods, coming on at five in the afternoon, and increasing in the night, so as to deprive him of sleep. The complaint had lasted five months. Purgatives of calomel, colocynth, and James's powder were given for several days, without any abatement of the pains. Mr. A. then directed ten grains of bark to be taken every two hours. In eight days the pains were greatly mitigated—and in a little more than a fortnight he was quite well.

When we consider that this patient took bark at the *rate* of half a drachm every six hours, the quantity was not very small in the aggregate, though the doses individually were minute.

*Case* 3. A woman, after exposure to wet and cold, on a voyage from Penzance to London, became afflicted with pains of a wandering nature for twelve months. After this they settled in her legs, and deprived her of rest, so that her health was considerably deteriorated. She had been under cure (or rather under treatment) at two dispensaries, without receiving any benefit. Mr. Allan, after the usual preliminary of purgation with calomel and colocynth, ordered the bark in *five grain* doses, with an equal quantity of carbonate of soda every two hours. The second night of this treatment was attended with sleep, and passed without pain. By a perseverance in the plan she got well.

*Case* 4. A poor woman had suffered, for several weeks, from a painful affection of one eye, remitting in the day, and coming on in severe paroxysms every evening, so as to deprive her of sleep in the night. A variety of remedies had been tried without effect. The eye, when Mr. A. first saw the patient, presented extreme vascularity of the conjunctiva with incipient ulceration of the cornea, great lachrymation, and symptoms of iritis. She had been put under the influence of calomel and opium for this last complaint, but without benefit. Mr. A. suspecting the inflammation to be of a rheumatic nature, ordered the bark in ten grain doses, with five grains of carbonate of soda every three hours—at the same time to take one grain of calomel with four of extract of colocynth every night. She was speedily cured by this plan.

We are tempted to give some particulars of the remaining case, for reasons which will presently appear.

*Case.* 5. A gentleman, 38 years of age, of nervous temperament, "and fond of his glass," who had long been subject to fits of syncope, and nervous tremors, was seized with pain of a peculiar character in the region of the right scapula, nor increased by pressure, and without any visible change of appearance. It was rather more troublesome in the night than in the day, but not always so. Purgatives and colchicum seemed rather to increase than diminish the evil. Mr. A. then tried his favourite remedy, bark; but here it failed completely. Leeches and fomentations afforded only temporary relief. The patient began to observe that the very act of talking about his complaint brought on the pain —so did shaving, and, at last, the sight of the barber excited the paroxysm. The carbonate of iron was now given, in doses of a scruple every six hours, increased, in a few days, to a drachm. Under this treatment the disease gradually declined, and soon completely disappeared.

Now let us see how far the observations in Mr. Allan's preliminary remarks apply to the cases which he has brought forward. We find the disease or diseases produced apparently by different causes—occurring in different sexes and constitutions—and occupying different textures of the body—in short, agreeing in no one character, except the prominent one, *periodicity*. Yet Mr. Allan, like a good practitioner, disregarding "those minute shades of difference in the features of diseases," is guided solely by one plain and obvious phenomenon, and applies the same remedy, in (almost always) the same dose, to five different patients. Thus we see that, at the bed-side, these refinements and minutiæ which are so much insisted on in the peroration, are thrown aside; and we are led by indications so plain that he who runs may read. One remark more on this point and we have done. In the fifth case, we do not find Mr. Allan (than whom there is not a better practitioner in London) with all his acumen and discrimination, able to select "the individual article precisely adapted to the peculiarities of his patient's case;" for he first tried the bark, in small doses, and was obliged to abandon it for carbonate of iron.

In respect to these small doses of bark, we are very much inclined to agree with Mr. Allan. Medicines, and especially the cinchona, are often placed in the same predicament as food. The good effects of both the one and the other depend, not so much on the quantity ingested as on the quantity digested. A great deal of bark and of carbonate of iron is often passed through the digestive canal unchanged. In this way, they may be given in large doses, without more benefit to the patient than if exhibited in much smaller ones. We think Mr. Allan's paper is calculated to afford some very useful hints to his brethren:—and, while we have ventured to question some points in his peroration, we are ready to accord the fullest meed of approbation to his practice.

---

### 26. SPONTANEOUS COMBUSTION.

M. Hellis of Rouen, relates a case of this kind in the April Number of the Journal de Médecine. The circumstances are these.

A woman named Soret, æt. 57, and very corpulent, had a most lively attachment for eau-de-vie, for which she was repudiated by her husband, who allowed her four francs a-week. On the night of the 31st December, having received her allowance, she repaired forthwith to the cabaret (*Anglicè*, gin-shop) where, in order to do all just honour to the new year, she got, if possible, more intoxicated than usual. The neighbours were not a whit surprised at her returning and making some stir at midnight; but, on hearing a loud crackling noise, they suspected fire; no unusual light however appearing, there was no inquiry made. In the morning, a woman having occasion to borrow something, went to Sorel's room, which she found filled with a dense smoke, though none of the furniture was on fire. The commissary of police was instantly called, and with him M. Hellis repaired to the spot in a short time afterwards. On entering the room they were struck with the strong empyreumatic smell and the thick smoke which presented themselves. The body was lying on the floor; little remained of it but the legs; part of the haunch, and the head. The chest, the belly and its contents, the trunk for the most part, and the upper extremities had disappeared. The bones of the fore-arm were calcined, so were the scapulæ, the pelvis was partly destroyed and contained only a shapeless mass of carboniferous matter. The clothes of the unfortunate woman were partly consumed. Strewed on the floor were the debris of animal carbon, some few pieces of wood charcoal, traces of blood near the head, and remains of a recent evacuation. The left hip was resting against a stool, and both were still burning. None of the furniture close by was damaged, a candle on the table, was extinguished, and nearly whole, and the cinders around were unctuous and highly fetid.

Such are the principal features of this case, and we must confess, it is a more satisfactory one than that related at page 286, No. 9, of this Journal. When we consider the quantity of combustible matter necessary to effect the destruction of the human body in its natural state, it is difficult, nay impossible, to account for the present case on any other supposition than that of spontaneous combustion. Scarcely were the clothes of the woman burnt; the furniture, with the exception of a stool in juxta-position with the body, remained unsinged, and yet the trunk, vertebræ and all, was utterly destroyed. The age of the woman, her immoderate love of eau-de-vie, her corpulency, and the circumstance of an ignited body, the candle, being present, make the case rather similar to the celebrated one of the Priest Bertholli. We think it probable that the woman, in her drunkenness, may have set fire to her clothes, and that this may have been the *origin* of the combustion, of course the progress and the result were owing to the state of the individual. It was, we believe, John Hunter, who, on dissecting a man who had been much addicted to the use of spirits, found the blood converted into a kind of oily matter. To notice the various theories that have been concocted to account for spontaneous combustion would fill many a quire. That which refers the occurrence to the extrication of hydrogen gas is the most plausible, and is in high favour in France, whilst, on this side the channel, little is said upon the subject, and not a few consider the whole a delusion.

